# Impact of Polymer Topology on Physical Aging of Thin Film Composite Membranes Based on PIM-1, cPIM-1, and Associated Blends

**DOI:** 10.1021/acs.macromol.4c02657

**Published:** 2025-03-13

**Authors:** Andrew B. Foster, Ming Yu, Mustafa Alshurafa, Peter M. Budd

**Affiliations:** Department of Chemistry, University of Manchester, Oxford Road, Manchester M13 9PL, U.K.

## Abstract

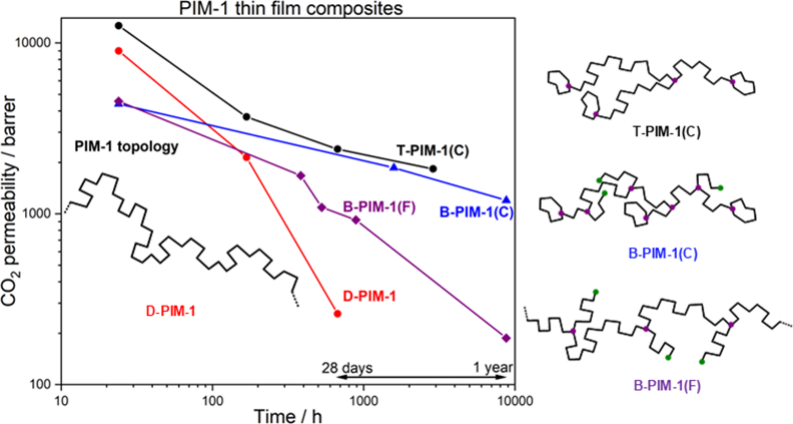

An overview is provided of the influence of polymer topology on the physical aging of PIM-1 thin film composite (TFC) membranes measured in gas permeation studies. Topologically distinct PIM-1 samples are compared first with each other, then in polymeric blends, and then with other literature. Both initial permeability (1 day) and long-term aging rates (up to 1 year) can be attributed to structural components present within the overall microstructure of the polymer. The rigidity and structural regularity of a predominantly disubstituted PIM-1 polymer proved to facilitate high initial CO_2_ permeability in TFCs followed by a rapid aging rate (β_P_ = 1.0) to produce an increasingly nonselective membrane over 28 days. By contrast, TFCs prepared from branched PIM-1 polymers, which have lower glass transition temperature, exhibit lower initial permeabilities followed by much slower aging rates, remaining highly selective for up to one year. Branched PIM-1 polymers which contain a greater proportion of small loop structures show a very slow aging rate (β_P_ = 0.22−0.25), whereas those with more open branched structure tend to exhibit a faster aging rate (β_P_ = 0.67−0.69). Thin film nanocomposite (TFN) membranes cast from blends of a disubstituted PIM-1 with colloidal network (CN)-rich PIM-1 fillers can completely halt permeability aging for up to one month but then subsequently resume aging at a faster rate (β_P_ = 1.8−2.8) to more than compensate. TFNs prepared from blending a branched PIM-1 polymer with a CN-rich Cardo-PIM-1 filler can produce better long-term aging performance (up to 1 year).

## Introduction

**Figure 1:**
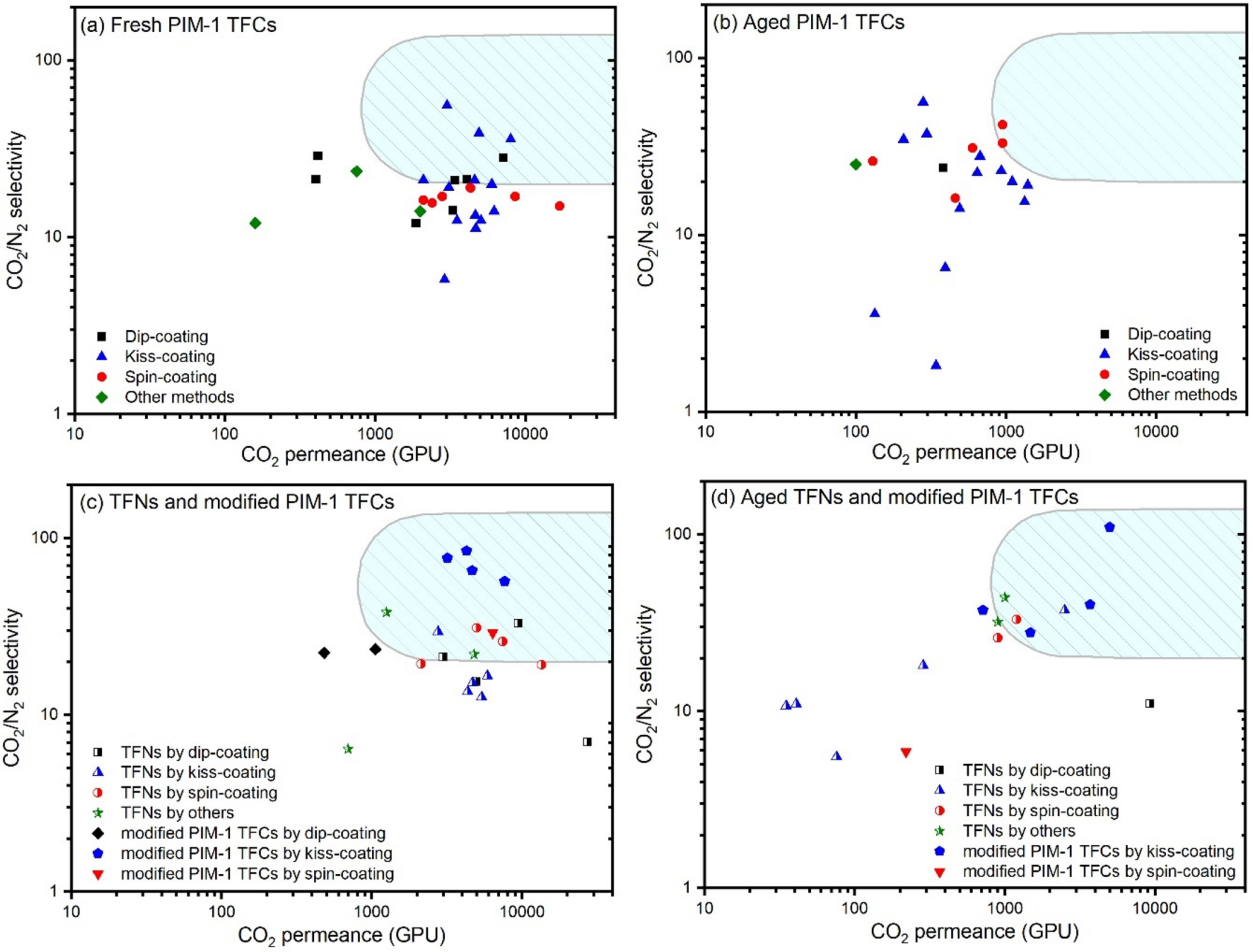
Ideal CO_2_/N_2_ separation performance of (a) fresh and (b) aged PIM-1 TFC membranes; (c) fresh and (d) aged PIM-1-based TFN membranes and modified PIM-1 TFC membranes (for example, cPIM-1) compared against the industrial favored range (shaded area) for post-combustion carbon capture suggested by Merkel et al.^25^

The archetypal polymer of intrinsic microporosity, PIM-1, has been extensively studied in membranes for gas separation applications since its initial report in 2004,^1^ with self-standing membranes exhibiting a large scatter in initial CO_2_ permeability (typically 3000–9000 barrer) and CO_2_/N_2_ selectivity (12–28) across different studies (Figure S1 and Table S1). There is a larger spread in membrane performance for polymers prepared under high temperature (150–160 °C) compared with low temperature (65–90 °C) polymerization conditions. After aging, most of the PIM-1 membranes depart further from the 2008 upper bound for the gas pair. Fabrication methods, post-treatment, and storage conditions are among the factors which can significantly affect film properties.^2^ When PIM-1 is processed into thinner selective layers (1–3 μm) as part of thin film composite (TFC) membranes, inherent differences in the polymer’s properties can be significantly magnified in physical aging and performance. Consequently, the reported gas separation performances of PIM-1 TFC membranes in the literature show an increased disparity in long-term performance. Gas separation performances of all reported fresh and aged PIM-1 TFC membranes in relation to CO_2_/N_2_ separation are summarized in Figure 1a,b and Tables S2 and S3.^3−24^ TFCs are characterized in terms of the mode of fabrication used, i.e., dip-, kiss-, or spin-coating or other methods, including bar- or spray-coating techniques. We define kiss-coating as a process in which contact between the coating solution and the support is controlled via surface tension, facilitated often by a roller coating technique. In general, when processed into TFC membranes, PIM-1 itself often struggles to meet the requirements for industrial post-combustion carbon capture,^25^ unless either blended with other polymers or compatible nanoscale fillers to create thin film nanocomposite (TFN) membranes or chemically modified, such as to carboxylated PIM-1 (cPIM-1) (Figure 1c).^3−8,12−19,22,23^ TFCs that are directly fabricated from solution via kiss- or dip-coating processes onto a porous support material often exhibit both decreased permeance and selectivity on longer-term aging (Figure 1b,d). Other techniques, such as spin-, bar-, or spray-coating, usually yield lower initial TFC performance and lack reporting of long-term aging in the literature. Introduction of a cross-linked poly(1-trimethylsilyl-1-propyne) (PTMSP) gutter layer on the support is one approach to avoid the loss of selectivity upon aging, but this often reduces overall permeance and is an extra step in the fabrication process.^9^ Coating on PTMSP involved kiss-coating a thin selective layer from a dilute mixed solvent solution of chloroform and trichloroethylene (0.5 wt %). Tiwari et al.^26^ have reported that spin-coated PIM-1 thin films (1 μm) from a lower boiling point solvent, chloroform, produce faster early stage aging rate than thinner films (200 nm) fabricated from *ortho*-dichlorobenzene. Generally, solution processing of PIM-1 for dip- or kiss-coating is carried out in chloroform, while the solvent used for cPIM-1 is tetrahydrofuran (THF).

Aging studies of PIM-1 thick films have indicated that initial free volume is the largest driver for free volume reduction.^27^ Modifications that introduce hydrogen bonds and cross-links can also impact diffusion of large gas molecules, improving selectivity. In addition to fractional free volume (FFV), the cohesive energy of the polymer chains (cohesive energy density, CED), related to the segmental motion of the polymer, has a role to play in terms of initial and long-term aging (changes in permeability, *P*), as outlined in eq 1;^28,29^ different polymer structures exhibit different aging rates.^30^ When comparing polymers with equivalent free volume, more rigid polymers, such as hexafluoro-substituted aromatic polyimides, show greater initial permeability.^31^ For a polymer with a very high glass transition temperature, gas transport is assumed to take place via movement from hole to hole along routes involving only minor segmental rearrangements.
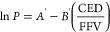
1

**Scheme 1:**
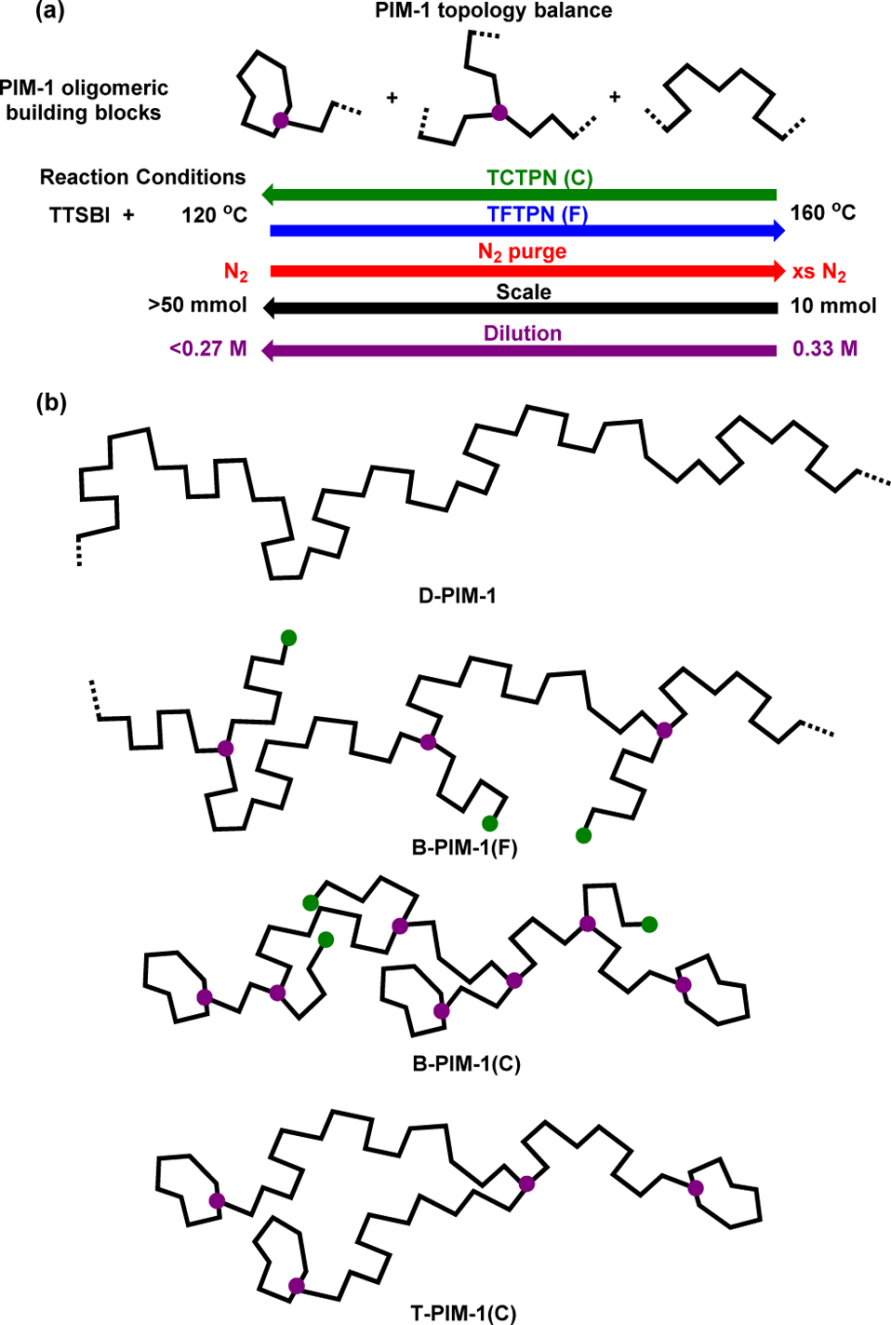
(a) Reaction Conditions Which Change the Proportions of Oligomeric Building Blocks (Small Loops, Branches, and Disubstituted Residue Structures) Formed during Early Stages of PIM-1 Step Growth Polymerizations; (b) Schematic Representations of Disubstituted Polymeric Structure (D-PIM-1), Different Types of Branched Polymer Structures Prepared from TFTPN and TCTPN [B-PIM-1(F or C, Respectively)], and Optimized Polymer Topology Structure (T-PIM-1)

Polymer topology is an important factor that significantly impacts the long-term performance of TFC membranes. We have reported variations in topology by varying polymerization temperature and level of N_2_ purging during synthesis of PIM-1 with a tetrachloro monomer (TCTPN, tetrachloroterephthalonitrile),^11,16^ instead of the conventionally used tetrafluoro monomer (TFTPN, tetrafluoroterephthalonitrile). In contrast to the standard linear structure formed by disubstitution reactions, monosubstitution at lower polymerization temperatures yields more branched PIM-1 structures, some of which can develop further into small loops or larger rings (Scheme S1). Reaction conditions (S3) best suited to change the distribution of oligomers forming in the early stages of step growth polymerization are summarized in Scheme 1. Schematic representations of structurally diverse forms of PIM-1 produced and discussed in this work are also drawn. The presence of branching can be characterized through proton nuclear magnetic resonance (^1^H NMR), indicated as smaller shoulder peaks adjacent to the main aromatic proton peaks. However, branching can lead to excessive insoluble network formation in extended time polymerizations in the absence of nitrogen purging, which affects the overall polymer sample processability as well as long-term membrane performance in TFC membranes. Comparing the number of branches (via ^1^H NMR or alternatively UV–vis spectrometry analysis) with the amount of chain ends present (via end group ^1^H NMR or chlorine analysis) allows estimation of loops within the polymer microstructure. Examples of these types of calculations are presented in Supporting Information, S4. Carrying out a nitrogen-purged PIM-1 polymerization with TCTPN at sufficient scale and dilution to marginally reduce the temperature ramp at which the reaction mixture is initially exposed, rising to a high set temperature of 160 °C, helps to create loop oligomers. These oligomers can act as chain terminators of otherwise predominantly disubstituted PIM-1 residue structures to limit the overall molar mass of the polymer formed (T-PIM-1(C) structure in Scheme 1). The incorporation of small loops in the overall PIM-1 polymer microstructure of a relatively low molar mass polymer (*M*_w_ = 62 kg mol^–1^) has been shown to enhance the long-term aging performance in TFC membrane applications.^16^ The densification of PIM-1 residue structures tethered within a small loop structure, or even within a larger ring structure formed later in a polymerization, would be expected to be very different to that envisaged from solely disubstituted PIM-1 residue polymer chains in the active layer of an aging TFC membrane. Molecular dynamics (MD) simulations have recently been utilized by Balcik et al.^32^ to explore physical aging of disubstituted PIM-1 structures. The effect of aging on density, *d*-spacing, FFV, and PSD was analyzed. MD simulations based around a wider range of topological types are underway to better understand the impact of topology on the relative balance between permeability and selectivity in aging thin films and will be the subject of a future publication.

This work draws together permeability and selectivity aging data of TFCs fabricated from a wide range of PIM-1 polymers and associated blends in Manchester and elsewhere, which have been reported in the literature since 2018. The PAN support material, on which the active layer is coated, is an important consideration in making comparisons. Since 2018, different batches of PAN support material, produced by Sepro Membranes (USA), have been utilized in our TFC work. PIM-1 TFCs discussed were generally fabricated via kiss-coating from chloroform solutions of the respective polymers or blend solutions onto these supports, unless highlighted otherwise.

## Results and Discussion

### TFC Performance of Topologically Distinct PIM-1 Polymers

**Figure 2:**
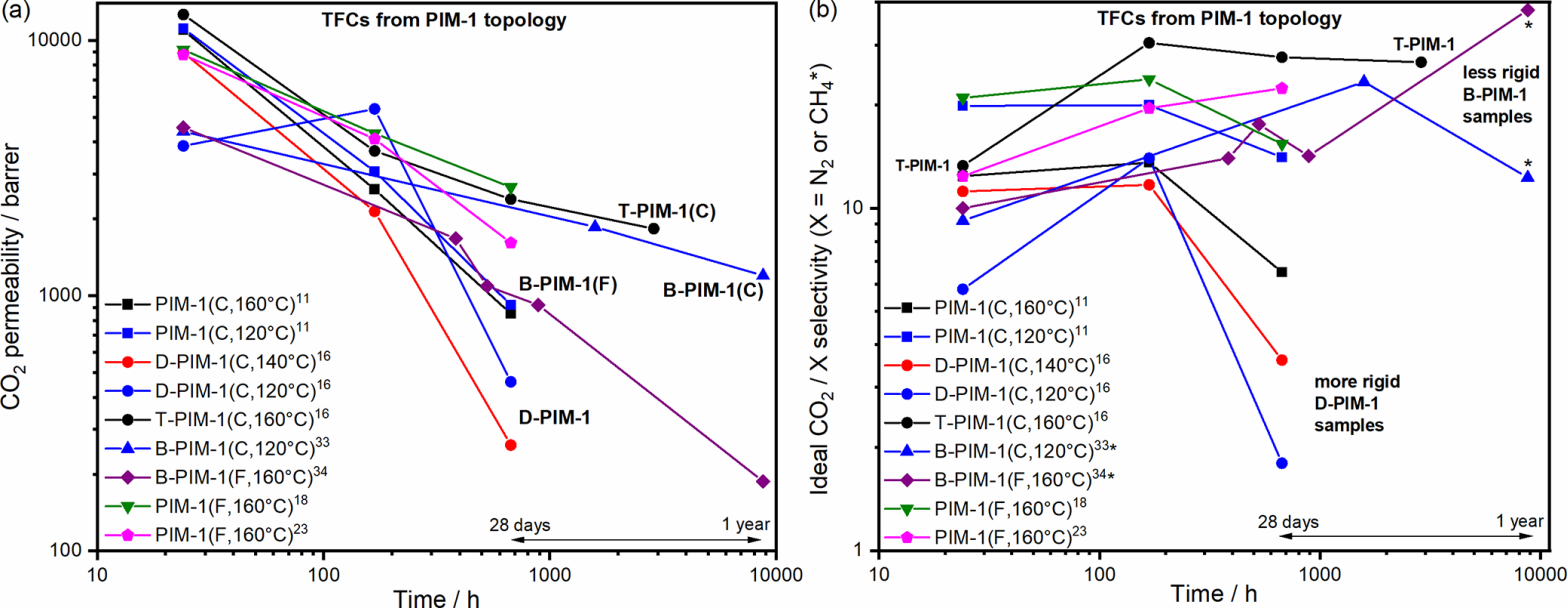
(a) CO_2_ permeability and (b) ideal CO_2_ selectivity aging data of PIM-1 TFCs fabricated from topologically different PIM-1 polymers in chloroform.

Comparisons between TFC aging performances obtained from a series of topologically different PIM-1 samples kiss-coated on similar PAN supports in Manchester are presented in Figure 2 (Table S6). The first six PIM-1 polymers were prepared from TCTPN (C) under different reaction temperature profiles (at set temperatures, 120–160 °C) and N_2_ purging conditions,^11,16,33^ as detailed in Table 1. PIM-1 produced under lower average temperature conditions (<160 °C) produce a complex topology range of polymeric structures, including branched, tadpole, cyclic structures, and some colloidal network (CN).^11^ Average heating temperatures, during fast, small-scale polymerizations (10 mmol) at set temperatures of 120 and 160 °C, defined as PIM-1 (C, 120 and 160 °C), were 112 and 121 °C, respectively. Lower molar mass polymers, coupled with higher CN contents, generally indicated improved gas separation performance in TFC membranes and particularly better aged performance in self-standing films. Relatively small differences were observed in CO_2_ permeability aging rates between these two polymers (Figure 2a), but much larger differences were observed in maintained selectivity upon aging (Figure 2b). The polymer prepared at a marginally lower average temperature which deviated more from the textbook disubstituted structure, via formation of branches and ring structures, holds on to its higher initial selectivity better than the polymer prepared at 160 °C. The aging comparisons between different PIM-1 polymers discussed in this section are presented sequentially across Figures S7–S9. Ring formation is an important aspect of polymerizations carried out with the tetrachloro monomer, and indeed polymerizations completed at a set temperature of 140 °C often exhibit solution viscosity behavior in size exclusion chromatography analysis which suggests richly cyclic polymer samples are obtained.^11,35^ This would only be the case if larger cyclic polymeric structures dominate the overall hydrodynamic volume across the polymer chain distribution.

**Table 1:** Synthesis and Characterization of PIM-1 Polymers and Colloidal Network (CN) Rich PIM polymers Used in Blending Which Were Solution Kiss-Coated into TFCs, Whose Physical Aging Are Presented in Figures 2 and 4

PIM-1 polymer^a^ or CN-PIM polymer^b^	scale/mmol	solvents	initial(/final) concentration/M	purging conditions	time/min	soluble polymer			colloidal network/%
						*M*_w_/kg mol^–1^	*D̵*_M_	branching/%	
PIM-1(C,160 °C)^11^	10	DMAc/DCB	0.33	N_2_	33	115.3	2.0	5.5	0.7
PIM-1(C,120 °C)^11^	10	DMAc/DCB	0.33	N_2_	81	94.8	2.3	3.0	7.8
D-PIM-1(C,140 °C)^16^	10	DMAc/DCB	0.33	xs N_2_	53	175.2	2.4	<2.0	0.8
D-PIM-1(C,120 °C)^16^	10	DMAc/DCB	0.33	xs N_2_	75	156.2	2.3	0	10.1
T-PIM-1(C,160 °C)^16^	50	DMAc/DCB	0.27	N_2_	40	62.4	2.1	6.1	6.6
B-PIM-1(C,120 °C)^33^	150	DMAc/DCB	0.33	N_2_	150	74.1	1.5	22.0	0
B-PIM-1(F,160 °C)^34^	30	DMAc/DCB	0.33	N_2_	38	85.0	2.0	11.7	0
PIM-1(F,160 °C)^18^	50	DMAc/toluene	0.27/0.21	N_2_	30	152.5	2.1	<2.0	0
PIM-1(F,160 °C)^23^	50	DMAc/toluene	0.27/0.21	N_2_	46	185.9	1.6	6.8	0
CN-PIM-1a(C,160 °C)^16^	10	DMAc/DCB	0.33	−	180	8.2	1.5	<2.0	85
CN-PIM-1b(C,140 °C)^16^	10	DMAc/DCB	0.33	−	180	23.4	1.4	<2.0	47
CN-Cardo-PIM-1(F,65 °C)^34^	25	DMF	0.17	N_2_	1440	38.5	5.7	n/a	70

a PIM-1 polymer name includes prefix description if a dominant polymer topology can be assigned to a particular sample: D-PIM-1 refers to a predominantly di-substituted polymeric sample, whilst B-PIM-1 refers to a heavily branched (>10 %) polymeric sample and T-PIM-1 refers to a particular sample with optimized topology which includes small loops (Figure 2). Information in brackets relates to the respective tetrahalo monomer (C = TCTPN or F = TFTPN) reacted with 5,5′,6,6′-tetrahydroxy-3,3,3′,3′-tetramethyl-1,1′-spirobisindane (TTSBI) in synthesis (3-fold molar excess of K_2_CO_3_ used in all cases) and the set point temperature for the respective polymerizations. The reaction temperature was measured in the case of the very large-scale polymerization (150 mmol), defined as B-PIM-1(C, 120 °C).^33^ The base was also only added once internal temperature reached 60 °C, whereas in all other polymerizations the base was present in reaction mixtures at commencement of heating up from room temperature to the set temperature.

b CN prefix indicates that a polymer sample contains a significant amount of colloidal network material. CN-PIM-1a and CN-PIM-1b were prepared in 3 h reactions of TCTPN (C) with TTSBI at 160 and 140 °C respectively in absence of nitrogen purge. In the case of CN-Cardo-PIM-1 polymerization, 50 mol % of TTSBI was replaced with 9,9-bis(4-hydroxyphenyl)fluorene (BHPF) in reaction with TFTPN at 65 °C for 3 days. Overall actual amounts of colloidal network (CN) present in blends with D-PIM-1 (C, 140 °C)^16^ or B-PIM-1(F, 160°C)^34^ are included in TFC aging information presented in Figure 4.

**Figure 3:**
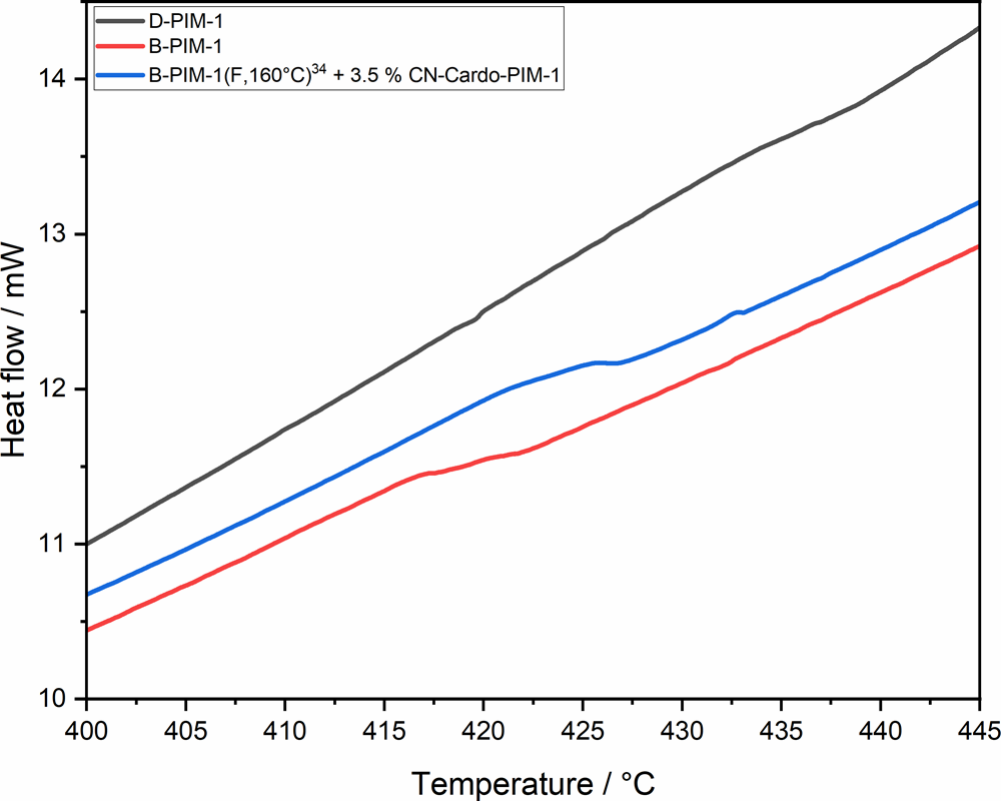
Enlarged high-temperature regions (400–445 °C) of DSC cooling cycles obtained for D-PIM-1, B-PIM-1, and B-PIM-1(F, 160 °C)^34^ + 3.5% CN-Cardo-PIM-1 blend. Further information on selected polymer samples and their complete DSC spectra is provided in S5 and Figures S2–S5.

Reaction conditions can be refined further to maximize the contrasts in topology between individual polymer samples.^16^ Polymerizations carried out at the same low scale (10 mmol) under higher N_2_ purging conditions at 140 °C produced a wholly disubstituted PIM-1 polymer (D-PIM-1) with minimal CN content, which when used in TFC membrane fabrication proved to age more rapidly than any other PIM-1 polymer and even lost selectivity on longer-term aging. By contrast, when a similar polymerization was completed at 120 °C, while the analyzed high molar mass soluble polymer content remained resolutely disubstituted, lightly branched structures were proposed to contribute to the 10.1% of CN content generated in the reaction. The presence of this amount of CN was sufficient to delay the commencement of rapid aging by 7 days, but very poor selectivity was again observed on longer-term aging of TFC membranes prepared for this predominantly disubstituted polymer, compared to that obtained for polymer samples with more complex topology balance, PIM-1 (C, 120 or 160 °C). Ultimately, predominantly disubstituted PIM-1 polymer chains in TFC membranes result in rapid permeability decline toward only 260 barrer and become barely selective in just over one month.

A larger-scale reaction (50 mmol) carried out, to a set point temperature of 160 °C starting with 20% excess of solvent, allowed the polymerization to proceed over a wider temperature profile to maximize topological balance, including the formation of small loops during the early stages of step growth polymerization [T-PIM-1(C, 160 °C)]. This polymer proved to exhibit better performance in TFC membranes, in both permeability and selectivity, than any others generated with the TCTPN monomer on longer-term aging. T-PIM-1 TFC membranes still exhibited permeability of 1830 barrer and ideal selectivity for CO_2_/N_2_ of 26.7 after 120 days. Furthermore, an even larger-scale polymerization (150 mmol) was carried out with TCTPN in which the reaction temperature was maintained at 120 °C throughout.^33^ This resulted in a polymer which contained a much higher level of branching at 22%, defined as B-PIM-1(C, 120 °C).^33^ The low polymerization temperature would be expected to facilitate some extra loop formation between some branches in the early stages of the step growth reaction. The comparatively low average number of end groups (*e* = 10 directly from chlorine content in elemental analysis) determined compared to branch points (aromatic proton NMR analysis) for this particular PIM-1 polymer would support the presence of at least 6 small loops within the average polymer chain microstructure. TFC membranes cast from this branched polymer exhibited a much lower initial permeability but then proceeded to age at a much slower rate than other polymer samples; it still exhibited CO_2_ permeability of 1200 barrer, and impressive CO_2_/CH_4_ selectivity, after one year. It should be noted that the overall CO_2_ permeability aging rate (1–365 days) also mirrored that observed for the T-PIM-1 polymer in the later stages of its aging (7–120 days). This contrast is attributed to the greater propensity to form small tadpoles or loops in the early stages of step growth with the chloro monomer, which acts as a chain terminator to the overall molar mass increase. The generally much improved aging for branched PIM-1 prepared from TCTPN is tentatively proposed to be due to the increased concentration of these ring structures within these polymers. A small polymeric loop or cyclic structure has much less freedom to rearrange and densify than an open linear or branched polymer chain in a thin film.

Generally, higher levels of branching are obtained by adjusting the reaction scale or rate of temperature increase.^19,35^ High levels of branching have been observed in some PIM-1 polymerizations prepared with TFTPN (F), for example, B-PIM-1(F, 160 °C).^34^ This relatively small-scale 30 mmol reaction with fluoro monomer proved to produce a relatively highly branched PIM-1: branch unit per every 9 disubstituted residues. This level of branching also provides added segmental mobility, yielding an initial low permeability for the TFCs. The TFCs proceeded to age relatively slowly, but not at the same level as polymers which are known to be richer in loops. Essentially, these TFCs took one year to reach the same level of permeability observed for D-PIM-1 TFCs within just over 1 month. The CO_2_ permeability of TFC membranes prepared from this branched polymer fell to around 190 barrer after one year of aging. Generally, PIM-1 polymerizations carried out with TFTPN (F) result in higher molar mass polymers than those produced with TCTPN (C) under equivalent conditions. For example, a diluted 50 mmol reaction with TFTPN monomer resulted in a high molar mass polymer (*M*_w_ = 153 kg mol^–1^) with minimal evidence of branching, defined as PIM-1(F, 160 °C).^18^ However, the aging profile of TFCs fabricated from this polymer more closely matched that of small loop-rich polymer (T-PIM-1), which suggests that even reaction solution dilution with fluoro monomer is sufficient to allow the small proportion of branches present to exist as part of small loop structures. A slightly more branched polymer prepared under similar initial conditions, PIM-1(F, 160 °C),^23^ but in which additional extra solvent was added much later in an overall longer polymerization, produced TFCs which tended to more closely follow the aging associated with the more heavily branched B-PIM-1(F, 160 °C).^34^

The inverse relationship between permeability and selectivity does not always hold on aging of TFCs prepared from PIM-1 and PIM-1 mixtures in chloroform; very large falls in permeability upon aging (Figure 2a) are often not compensated by rises in selectivity (Figure 2b). The most rapidly aging PIM-1 TFC membranes (1–3 μm active layer) densified and lost permeability at least five times more rapidly than self-standing films (80–110 μm).^36^ Interfacial interactions, either between rigid polymer chains with the underlying PAN support or perhaps within an interpenetration layer, may play a role in aged selectivity. If tension leading to selectivity fall develops solely between densified polymer, this would have been observed previously in the aging of very thin films directly formed in the absence of support material.^26^ Loss of both permeability and selectivity on aging has been observed with very thin selective layers (<100 nm) spin coated onto anodic aluminum oxide substrate.^24^

**Scheme 2:**
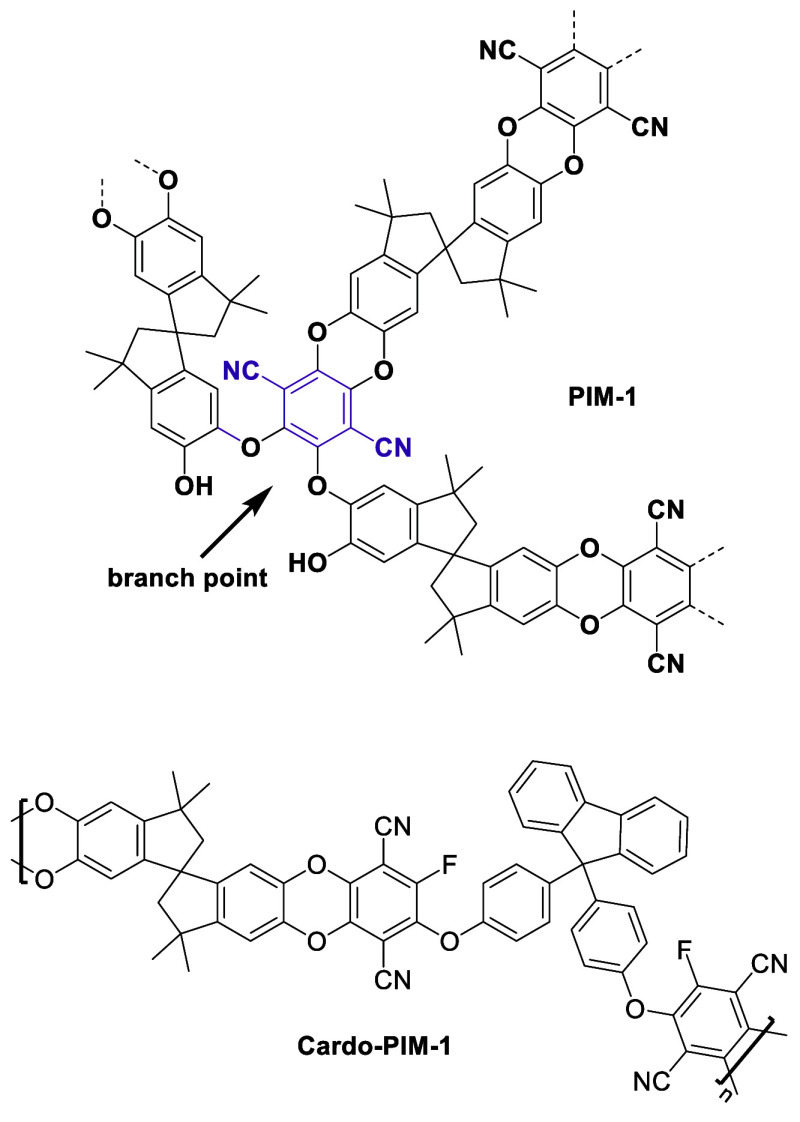
Monosubstituted Linkages within Branched PIM-1 and Cardo-PIM-1 Structures Which Introduce Greater Segmental Mobility into Their Respective Polymeric Chains

The initial TFC permeability obtained, on similar PAN supports and the same coating conditions, for each PIM-1 polymer sample appears to be largely governed by the respective amounts of branching present. More highly branched PIM-1 polymers exhibit 2-fold lower permeabilities than predominantly disubstituted PIM-1 polymer samples. Branches, owing to their connectivity, introduce points of greater mobility within the overall polymer chain structure of PIM-1 (Scheme 2). The rigidity of a disubstituted PIM-1 polymer structure might be a reason for failing to obtain a glass transition temperature in conventional DSC (differential scanning calorimeter) analysis,^31^ producing very small change in heat capacity of polymer (Δ*C*_*P*_) transitioning between glassy and rubber states. Only relatively recently, fast scanning DSC analysis has recorded a *T*_g_ of 442 °C for this PIM-1 structure.^37^ By contrast, a heavily branched (11%) PIM-1 polymer (B-PIM-1) sample is measurable by conventional DSC analysis, yielding a distinctive transition at a lower temperature of 419 °C, as shown in Figure 3. For comparison, equivalent DSC analysis of a predominantly disubstituted PIM-1 (D-PIM-1) polymer is largely featureless, with only a hint of a transition toward 440 °C. An increase in Δ*C*_*P*_ can be attributed to the decreased rigidity of a branched PIM-1 polymer structure.^31^ Literature indicates that the introduction of branching leads to glass transition temperature decrease from 266 °C for linear polyimide down to 222 °C for amino-terminated and 231 °C for anhydride-terminated hyper-branched polyimides.^38^ It is proposed that the overall macroscopic mobility of the polymer chains governs the initial disordered packing of the polymer chains in the formed thin active layer and thus early permeability (differences evident at 1 day of aging). Aging after this point is thus predicted to be governed by how individual polymer chains interact with each other. Lower initial permeabilities were generally observed for TFCs prepared from branched PIM-1 polymers.

As has been discussed elsewhere with regards to thick PIM-1 films,^36^ decreases in permeability with time are more or less linear on a log–log scale (Figure 2a). The impact of physical aging on CO_2_ permeability (*P*) can be approximated using eq 2
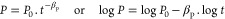
2where *P*_0_ is an initial permeability where *t* > 0, and β_P_ has the physical meaning of a permeability aging rate constant.

Aging rates in β_P_ (−∂[log(*P*/barrer)]/∂[log(*t*/h)]), observed between aging time intervals across TFCs fabricated from a range of PIM-1 polymers, whose topology is known, were determined (Table 2). D-PIM-1 TFC membranes exhibit β_P_ values of at least 1.0 in aging over 28 days, compared to 0.24 reported for thick self-standing films.^36^ It should be noted that the thick films were cast from a PIM-1 polymer synthesized in DMF at 65 °C for 72 h; low temperature polymerizations do tend to contain branched structures.^39^ Predominantly disubstituted PIM-1 polymer contains fewer side chains (from branching) and thus would be predicted to pack down and densify relatively rapidly as a thin film. By contrast, heavily branched polymer chains need to overcome side chain interactions before the respective backbones of neighboring chains would come into closer contact with each other in later densification. Two different, slower aging rates, β_P_ around 0.22–0.25 or 0.67–0.69, are evident for TFC membranes prepared from branched PIM-1 polymers synthesized from TCTPN or TFTPN, respectively. Branched polymers prepared with TCTPN are lower in molar mass as some chain ends form small loops to cut down the molar mass of the final polymer, whereas with TFTPN monomer the branched oligomers tend to link up further to build up to higher molar mass polymers. The PIM-1 polymer with optimized topology (T-PIM-1), which contains small loops combined with relatively long sections of disubstituted PIM-1 residues, exhibits two distinctive aging rates. Early faster aging between 1 and 7 days (β_P_ ≈ 0.6) would appear to be driven mostly by disubstituted PIM-1 sections densifying, before exhibiting from 7 to 120 days a much lower aging rate, β_P_ ≈ 0.25, which in this case must be attributable to small loops within the structure.

**Table 2:** CO_2_ Permeability Aging Rates (β_P_) for PIM-1 Polymers (Figure 2a) during Different Time Intervals

polymer	aging period/days	aging rate, β_P_
PIM-1(C,160 °C)^11^	1–28	0.77
PIM-1(C,120 °C)^11^	1–28	0.74
D-PIM-1(C, 140 °C)^16^	1–7	0.74
	7–28	1.52
D-PIM-1(C, 120 °C)^16^	1–7	–0.17
	7–28	1.78
T-PIM-1(C, 160 °C)^16^	1–7	0.63
	7–120	0.25
B-PIM-1(C,120 °C)^33^	1–365	0.22
B-PIM-1(F, 160 °C)^34^	16–365	0.67
PIM-1(F, 160 °C)^18^	1–28	0.37
PIM-1(F, 160 °C)^23^	1–7	0.39
	7–28	0.68

### TFN Performance of PIM-1 Blended with Network-Rich PIM Polymers

**Figure 4:**
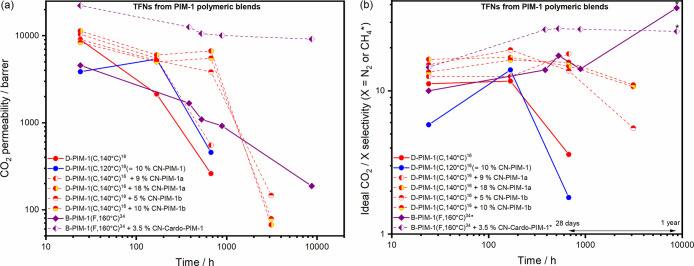
(a) CO_2_ permeability and (b) ideal CO_2_ selectivity aging data of PIM-1 TFCs (lines) and their polymeric blended TFNs (dashed lines) fabricated from chloroform solutions (3% w/v).

TFN membranes prepared via kiss-coating from blends of PIM-1 with structurally compatible PIM network nanoparticulates have also proved important to understand the factors governing aging in thin films, as presented in Figure 4 (Table S7). The aging comparisons for the three types of blends discussed in this section are presented sequentially in Figures S10–S12. When a fully disubstituted PIM-1 polymer [D-PIM-1(C, 140 °C)]^16^ was blended with CN-rich PIM-1 versions (termed CN-PIM-1a or CN-PIM-1b in Table 1), permeability decline could be arrested for up to 28 days with only a 10 wt % loading of the more densely cross-linked CN from CN-PIM-1b. TFC membranes prepared from the D-PIM-1 polymer blended with these CN versions then exhibit much faster resumed aging rates (β_P_ around 1.8–2.8) after overcoming the temporal halt on aging that the rigid CN initially provided to the overall blend structure. It should be noted that another predominantly D-PIM-1 polymer sample synthesized at a lower set temperature of 120 °C, which also in itself generated 10 wt % CN, exhibited a very similar aging profile to that of the pure D-PIM-1 polymer subsequently blended with 9 wt % colloidal content from CN-PIM-1a.

TFC membranes prepared from a heavily branched PIM-1 polymer, B-PIM-1(F, 160 °C),^34^ exhibited a lower initial permeability, followed by a much lower aging rate [β_P_ (16 to 365 days) = 0.67] for up to one year. When this type of branched polymer was then blended with low levels of a CN-rich form of Cardo-PIM-1 (CN-Cardo-PIM-1 in Table 1), it resulted in TFC performance in which prolonged high CO_2_ permeability of 9140 barrer and ideal CO_2_/CH_4_ selectivity of 26 were still evident after one year. The greater stability of this blend was proposed to be due to the formation of a semi-interpenetrating polymer network between the portion of CN-Cardo-PIM-1 and the branched PIM-1 polymer, as mentioned above. The structure of Cardo-PIM-1, based on the monomers used, is presented in Scheme 2. The high colloidal content present indicated that some of the fluorine atoms are consumed in further reactions to create network structures. Both of the polymers which made up the blend in this case are richer in monosubstituted linkages than are normally reported for aromatic nucleophilic disubstitution reactions associated with PIM-1. The CN-Cardo-PIM-1 polymer also exhibited a conventionally measurable *T*_g_ of 414 °C (Figure S4). The increased capacity for hydrogen bonding and added segmental mobility of the branched PIM-1 polymer chains would appear to facilitate better long-term compatibility with the more rigid CN Cardo-PIM-1. A higher glass transition temperature of 423–426 °C was obtained from a self-standing film prepared from B-PIM-1(F, 160 °C)^34^ containing 3.5 wt % CN from CN-Cardo-PIM-1 polymer (Figure 3).

### TFN Performance of PIM-1 Blended with Other Fillers

Some earlier TFN studies in Manchester in 2018, which examined the performance of concentrated blends of carbonized hyper-cross-linked polystyrene, c-HCP, with PIM-1, exhibited impressive long-term permeability but low selectivity on a similar PAN support.^6^ This dip-coated work was completed before PIM-1 polymer topology was fully understood; however, the base PIM-1 polymer used in that work exhibited similar aging performance [β_P_ (30 to 90 days) = 0.69] and selectivity to that expected for a branched PIM-1 polymer synthesized from TFTPN (Table S8). Branched polymers were being produced in Manchester in this period as a consequence of extra amounts of solvent being added simply to maintain mixing during larger-scale polymerizations.^35^

**Figure 5:**
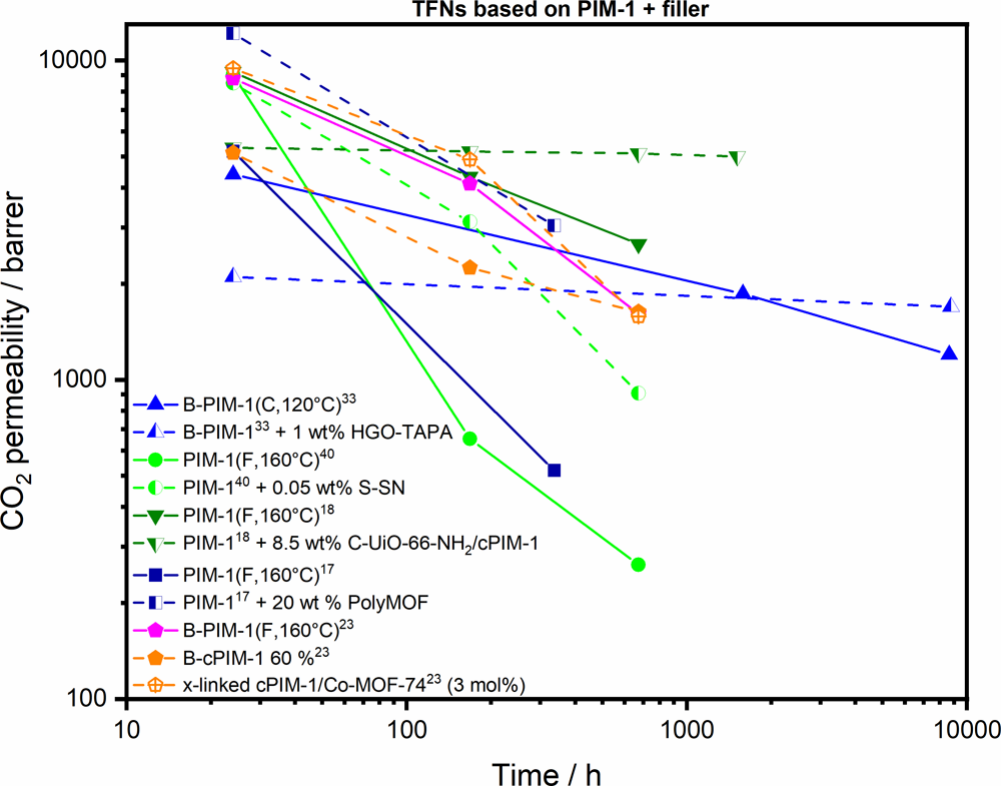
CO_2_ permeability aging data of TFC membranes prepared from PIM-1 polymers (lines) and corresponding TFN membranes including various fillers (dashed lines).

**Figure 6:**
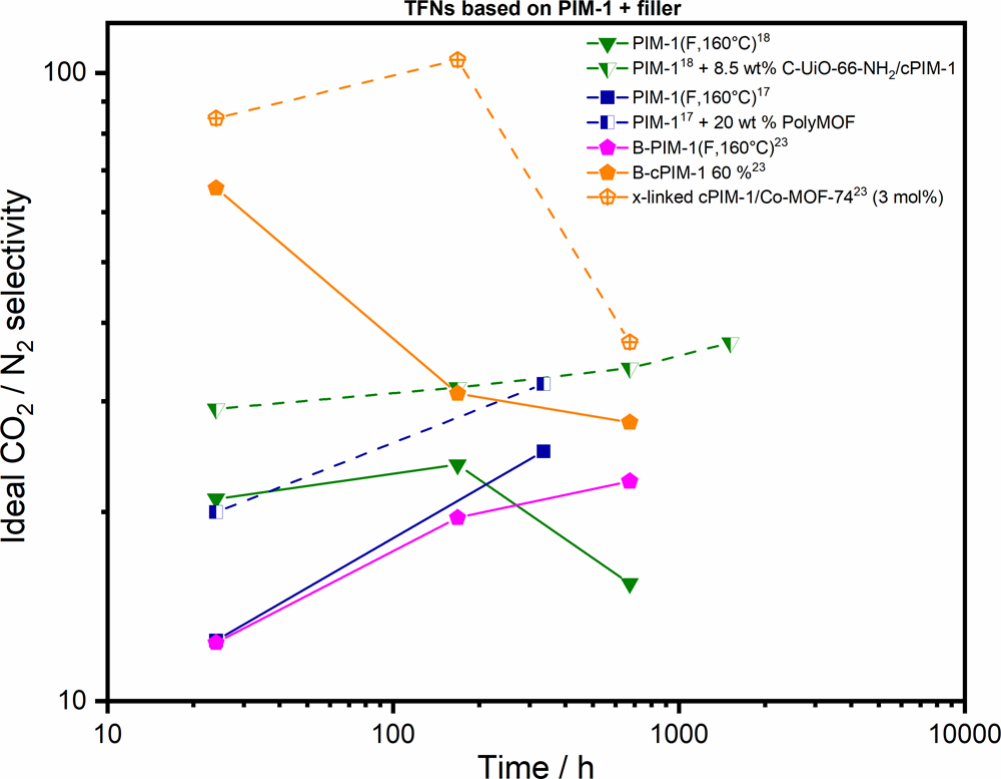
Ideal CO_2_/N_2_ selectivity aging data of TFC membranes from PIM-1 polymers (lines) and corresponding TFN membranes (dashed lines) including various fillers.

**Figure 7:**
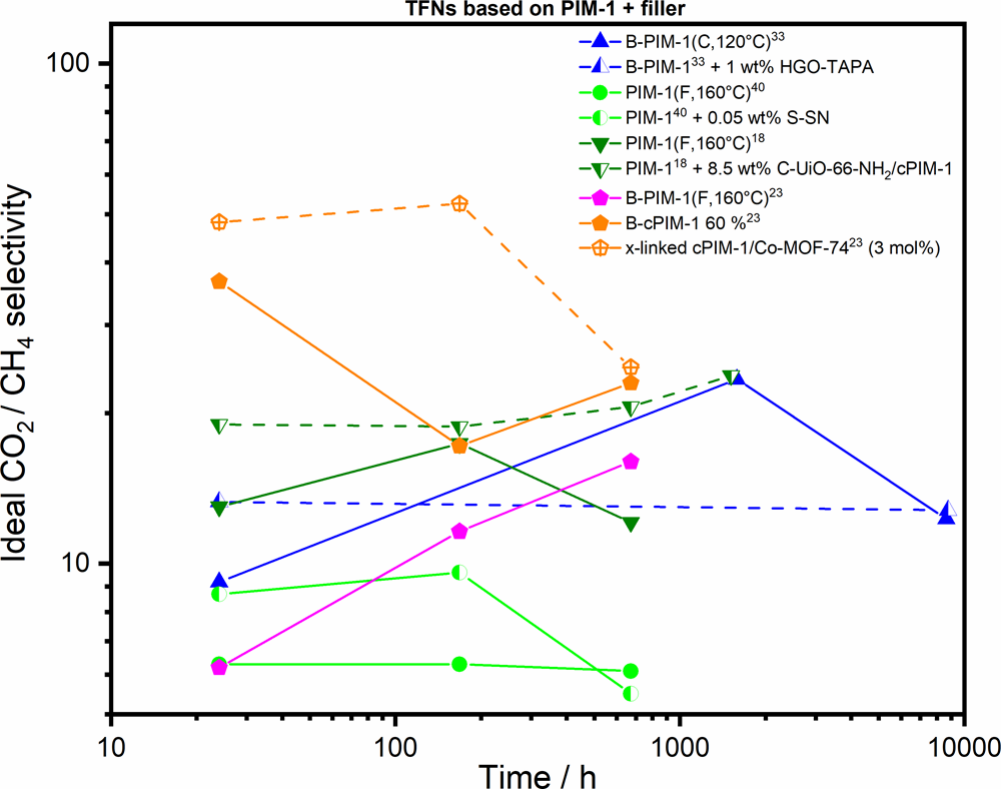
Ideal CO_2_/CH_4_ selectivity aging data of TFC membranes from PIM-1 polymers (lines) and corresponding TFN membranes (dashed lines) including various fillers.

TFN membranes prepared via dip-, kiss-, or bar-coating from blends of PIM-1 with modified 2D fillers or inorganic-based fillers have also been much studied^17,18,23,33,40^ to try to improve the permeability and selectivity balance within aged thin films; examples are presented in Figures 5–7 (Table S8). Some good improvements have been reported in these blending studies relative to the base PIM-1 polymer used in each case. These plots again emphasize the need to always reference blending studies against the base polymer sample. Differences in PIM-1 polymer, support material, and coating procedure used may contribute to differences observed in initial permeability. Some of these base polymers exhibit CO_2_ permeability aging behavior akin to that observed for a predominantly disubstituted polymeric (D-PIM-1) backbone, while others show aging more associated with more topologically diverse polymers. It should be noted that one of the best PIM-1 samples used in blending was prepared in diluted 50 mmol scale with TFTPN at 160 °C and exhibited minimal amount of branching but aged slowly in permeance over 28 days (Figure 5).^18^ As discussed earlier, the high initial permeability is as expected for predominantly disubstituted polymer structure, but the slower subsequent aging, if not attributable to excessive branching in this case, would suggest the presence of other topological features, such as loops, expected to be generated in the early stage of polymerization, due to excess amount of solvent present (Table 1). The selectivity performance, however, seems to be governed by the prevalent disubstituted residues, as it started to decrease after only 28 days (Figure 6). It should also be qualified that, in this case, TFC membranes of both base PIM-1 polymer and blended TFNs were prepared from kiss-coating a more concentrated polymer solution of 4.5% w/v in chloroform/THF mixed solvent system at a ratio of 9:1 in volume. TFNs of this polymer blended with 8.5 wt % C-UiO-66-NH_2_/cPIM-1 exhibited stable permeability and improved selectivity behavior on aging up to 60 days.

An example is also included of a moderately branched PIM-1 polymer sample, 'PIM-1(F, 160 oC),^40^ which produced TFC membranes whose overall permeability aging profile was very poor (Figure 5). In this case, a 30 mmol scale polymerization, with TFTPN, was carried out, which resulted in a 5% branched PIM-1 polymer. The poorer performance may be attributed to the relatively small-scale reaction, with no excess solvent added, which produced a lower level of branches that did not provide much opportunity for loop formation in the early stages. This is also reflected in much lower ideal CO_2_/CH_4_ selectivity than other PIM-1 polymer samples (Figure 7). TFNs fabricated from blending this polymer with 0.05 wt % porous silica nanosheets functionalized with sulfonic acid groups (S-SN) exhibited a slower permeability decline and no real improvement in selectivity performance.

### TFC Performance of Carboxylated PIM-1

**Figure 8:**
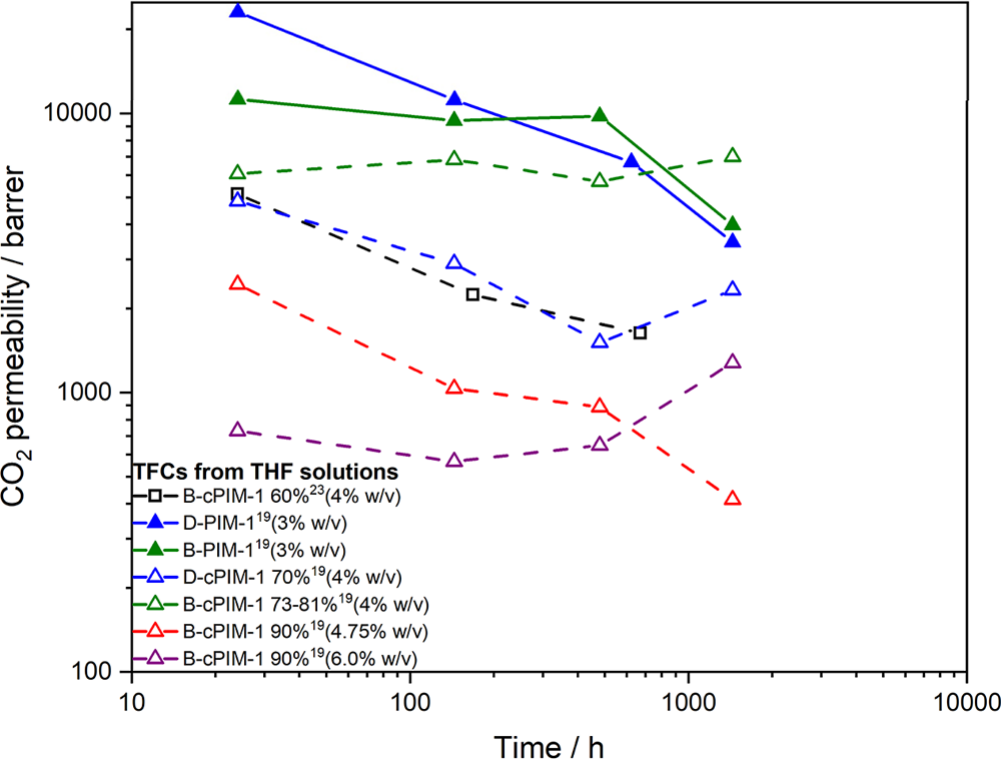
CO_2_ permeability aging data of TFC membranes prepared from PIM-1 (lines) and cPIM-1 (dashed lines) polymers fabricated from THF solutions.

**Figure 9:**
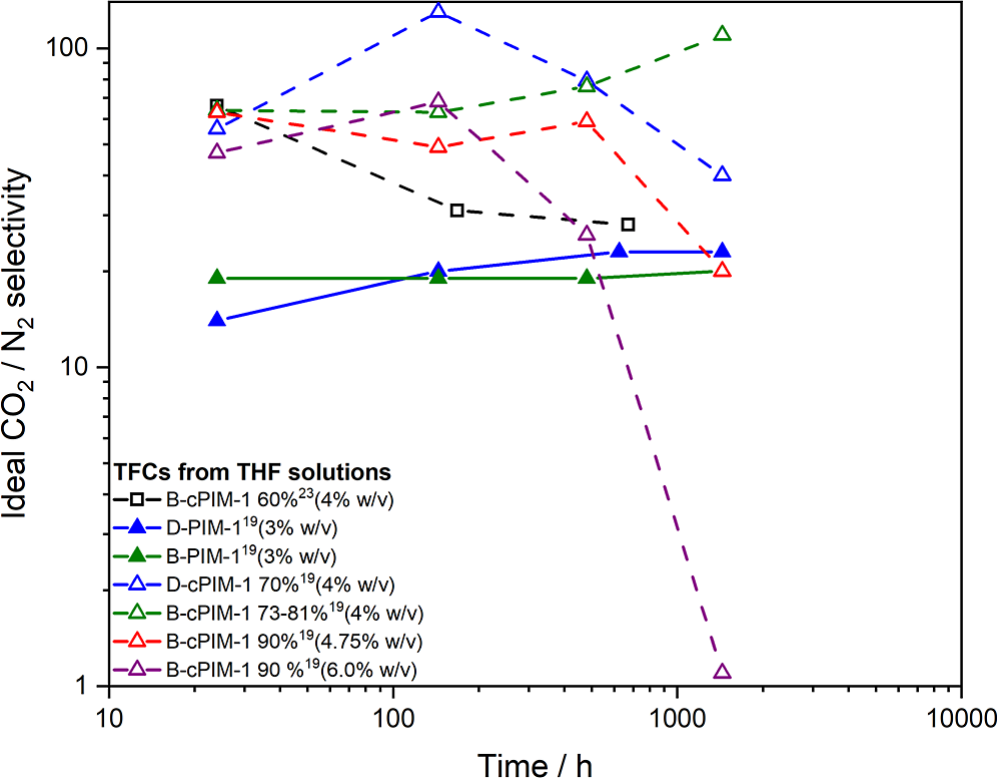
Ideal CO_2_/N_2_ selectivity aging data of TFC membranes from PIM-1 (lines) and cPIM-1 (dashed lines) polymers fabricated from THF solutions.

Carboxylated PIM-1 (cPIM-1) is one of the most studied types of modified PIM-1 for gas separation performance and has been fabricated into TFCs on Sepro PAN supports.^19^ cPIM-1 polymer is only soluble in THF, so TFCs were fabricated from this solvent for both PIM-1 and cPIM-1 for comparison purposes (Figures 8 and 9 and Table S9). PIM-1 TFCs fabricated from THF solutions also typically exhibit higher initial permeability for a predominantly disubstituted PIM-1 (D-PIM-1, 3.7% branched)^19^ polymer, than a more branched (B-PIM-1, 6.5% branched)^19^ polymer; the same trend as observed from chloroform. Both of the PIM-1 polymers included were synthesized under dilute initial conditions, at 50 mmol scale, which enhances the potential for formation of loops, with a slightly low temperature ramp profile producing a more branched PIM-1 polymer. Beyond 20 days, relatively thick active layer (3.6–3.7 μm) TFCs from both polymers show similar patterns of increased permeability aging (β_P_ ≈ 0.8), while at least maintaining their selectivities for up to 60 days. Much higher ideal CO_2_/N_2_ selectivities are observed initially for cPIM-1 TFCs with different levels of hydrolysis than for PIM-1 TFCs, but most of this large enhancement over the parent polymer has been shown to disappear due to increased plasticization in mixed gas testing.^19^ However, how the permeability and ideal selectivity change with aging for cPIM-1 TFCs, with increasing levels of hydrolysis and solution concentration, is still of interest as they give insight into the impact of excessive hydrogen bonding on the overall performance of thin film membranes. TFCs fabricated from a concentrated solution of 90% hydrolyzed, branched PIM-1 densifies initially to yield the lowest permeability of the series and eventually loses all selectivity after 60 days aging. Performance with lower levels of hydrolysis, between 60 and 80%, does suggest levels of interactions which help to maintain much better balance between permeability and selectivity in longer-term aging.

## Conclusions

PIM-1 polymerizations from TCTPN produce greater topological diversity, which when fabricated into TFC membranes exhibit slower permeability aging rates compared to TFTPN-derived polymers and maintain their selectivity better upon long-term aging. Polymerization conditions can be used to optimize the topological balance further through careful use of reaction scale, solution concentration, set point temperature, and nitrogen purging conditions. Branching occurs in polymerizations from both TFTPN or TCTPN, increasing the overall segmental mobility within the polymer chains, which is evidenced by a lower measurable *T*_g_ than observed for a predominantly disubstituted PIM-1 polymer. This translates into TFC membranes that exhibit a lower initial permeability (1 day), potentially attributed to added mobility allowing the respective branched chains to collapse and fill space initially more efficiently, compared to the rigidity of disubstituted polymer chains, which maintain a higher free volume and permeability. Aging beyond this point depends on the proportion of branches that exist as part of small loop structures, which are predicted to densify differently than an open branch in the polymer microstructure. PIM-1 polymers rich in small loops, derived from TCTPN, exhibit the slowest aging rates (β_P_ = 0.22–0.25), whereas branched polymers derived from TFTPN with less loops typically exhibit significantly faster aging rates (β_P_ = 0.67–0.69). Notably, it has also been observed that diluted (0.21–0.27 M), high set point polymerizations (160 °C) carried out with TFTPN, when scaled up sufficiently (≥50 mmol) to maximize conversion of branches into loops in early stages of polymerization, can exhibit comparable slower physical aging profiles.

By contrast, predominantly disubstituted polymer chains densify very rapidly from their initial high free volume state (β_P_ > 1.0 between 1 and 28 days) and lose their selectivity in longer-term aging. In addition, if a disubstituted PIM-1 polymer is blended with a CN-rich, disubstituted PIM-1, TFC permeability aging can be halted for up to 1 month before resuming at an even faster aging rate (β_P_ = 1.8–2.8) to compensate.

Longer-term selectivity of PIM-1-based TFCs or TFNs is largely governed by the proportion of monosubstituted linkages present within the overall polymeric structures or blends. Branched polymers are shown to interact better with a monosubstituted rich polymeric filler (CN-Cardo-PIM-1) to provide much better long-term balance of permeability and selectivity.

Controlled hydrolysis (60–80% conversion) of PIM-1 polymers, with already optimized topology, to cPIM-1 can deliver improvements in long-term aging in fabricated TFCs and should show increased potential interactions in blending to create TFNs, which may address the plasticization issues which often accompany thin film PIM membranes.

## Data Availability

Data supporting this study are available within the article and the Supporting Information.

## Supporting Information Available

Gas permeation performance of self-standing PIM-1 films from literature, overall single gas testing data of PIM-related TFC and TFN membranes with aging from literature, PIM-1 syntheses and nomenclature overview, estimation of loops in average PIM-1 polymer chain from polymer analysis, description of fabrication (kiss-coating technique), DSC analysis of PIM polymers, and tables of single gas testing data (PDF)

## Abbreviations

PIM: polymer of intrinsic microporosity
TFTPN: tetrafluoroterephthalonitrile
TCTPN: tetrachloroterephthalonitrile
TTSBI: 5,5′,6,6′-tetrahydroxy-3,3,3′,3′-tetramethyl-1,1′-spirobisindane
PAN: polyacrylonitrile
TFC: thin film composite
TFN: thin film nanocomposite
CN: colloidal network
