# Supplementary material for: Impact of Polymer Topology on Physical Aging of Thin Film Composite Membranes Based on PIM-1, cPIM-1, and Associated Blends
Source: Macromolecules. 2025 Mar 13;58(8):4289–99. doi: 10.1021/acs.macromol.4c02657 (PMC12020468; doi:10.1021/acs.macromol.4c02657)
Supplement: Supplementary file 1 — ma4c02657_si_001.pdf [file ma4c02657_si_001.pdf]

Supporting information

Impact of polymer topology on physical aging of thin film composite membranes based on PIM-1, cPIM-1 and associated blends

Andrew B. Foster*, Ming Yu, Mustafa Alshurafa and Peter M. Budd*

*corresponding authors, email: andrew.foster@manchester.ac.uk, peter.budd@manchester.ac.uk

Department of Chemistry, School of Natural Sciences, The University of Manchester, M13 9PL Manchester, U.K.

Table of Contents

S1. Gas permeation performance of self-standing PIM-1 films. 1

Figure S1. Selection of ideal CO_2/N_2 separation performances of (a) fresh and (b) aged self-standing PIM-1 film membranes reported in the literature.[1-14] 1

Table S1. Reported ideal CO_2/N_2 performance of self-standing PIM-1 membranes (fresh and aged) prepared from different polymers synthesized under the outlined polymerization conditions. [1-14] 2

S2. Gas permeation performance of PIM-1 thin film composites (TFCs), PIM-1 based thin film nanocomposites (TFNs) and modified PIM-1 TFCs fabricated by different methods. 3

Table S2. Reported ideal CO_2/N_2 performance of PIM-1 TFC membranes (fresh and aged) prepared via dip-, kiss-, spin- coating or other methods (presented in Figures 1(a) & 1(b)). 3

Table S3. Reported ideal CO_2/N_2 performance of PIM-1 TFN membranes or modified PIM-1 TFC membranes (fresh and aged) prepared via dip-, kiss-, spin- coating or other methods (presented in Figures 1(c) & 1(d)). 5

S3. PIM syntheses and nomenclature for polymers used in TFC and TFN membranes. 7

Scheme S1. PIM-1 polymerization. 7

S3.1. PIM-1 polymerizations (0.33 M). 8

Table S4. PIM-1 synthesis conditions for 0.33 M polymerizations presented in Table 1. 9

S3.2. Diluted PIM-1 polymerizations (0.27/0.21 M). 9

Table S5. PIM-1 synthesis conditions for 0.27 (/0.21) M polymerizations presented in Table 1. 10

S4. Estimation of loops in average PIM-1 polymer chain. 10

S4.1. Topology estimation for T-PIM-1(C, 160 °C)[16] polymer. 11

S4.2. Topology estimation for B-PIM-1(C, 120 °C)[33] polymer. 11

S5. DSC analysis of PIM-1 polymers. 12

Figure S2. (a) Full DSC trace and (b) expanded region of D-PIM-1 polymer. 13

Figure S3. (a) Full DSC trace and (b) expanded in region of defined glass transition of B-PIM-1 polymer. T_g = 419 °C. 14

Figure S4. (a) Full DSC trace and (b) expanded in region of defined glass transition of CN-Cardo-PIM-1 polymer. T_g = 414 °C. 15

Figure S5. (a) Full DSC trace and (b) expanded in region of defined glass transition of self-standing film prepared from B-PIM-1 blended with 3.5 wt % CN-Cardo-PIM-1 polymer. T_g = 423-426 °C. .. 16

S6. Fabrication of TFCs and TFNs via kiss-coating procedure. 17

S6.1. PIM solutions used in TFC/TFN fabrication. 17

S6.2. Thin film composite membrane preparation (kiss-coating technique). 17

Figure S6. Thin film kiss-coating setup with a steel roller coater connected to a motor, steel tray for containing coating solution, and glass plates for supporting the tray to control the contact between PAN support and solution. 18

S7. Gas permeation tests. 18

S8. Thin film composite (TFC) performance of topologically distinct PIM-1 polymers. 20

Figure S7.(a,b) Comparisons discussed within TFC aging data fabricated from different PIM-1 polymers presented in Figure 2. 20

Figure S8.(a,b) Comparisons discussed within TFC aging data fabricated from different PIM-1 polymers presented in Figure 2. 21

Figure S9.(a-c) Comparisons discussed within TFC aging data fabricated from different PIM-1 polymers presented in Figure 2. 22

S9. Thin film composite (TFC) and thin film nanocomposite (TFN) performance of PIM-1 polymers blended with network rich PIM polymers. 23

Figure S10.(a-c) Comparisons discussed within TFC and TFN aging data fabricated from D-PIM-1 polymer and their blends with network rich, CN-PIM-1a polymer presented in Figure 4. 23

Figure S11.(a,b) Comparisons discussed within TFC and TFN aging data fabricated from D-PIM-1 polymer and their blends with network rich, CN-PIM-1b polymer presented in Figure 4. 24

Figure S12.(a,b) Comparisons discussed within TFC and TFN aging data fabricated from B-PIM-1 polymer and blend with network rich, CN-Cardo-PIM-1 polymer presented in Figure 4. 25

S10. Permeability and ideal selectivity aging of PIM-1 based TFC and TFN membranes prepared since 2018. 26

Table S6. Permeability and ideal selectivity aging performance of kiss-coated PIM-1 TFCs, presented in Figure 2. 26

Table S7. Permeability and ideal selectivity aging performance of kiss-coated PIM-1 TFCs and TFNs of blends with network-rich PIM polymers, presented in Figure 4. 28

Table S8. Permeability and ideal selectivity aging performance of PIM-1 TFCs and TFN blends with other fillers. 30

Table S9. Permeability and ideal selectivity aging performance of PIM-1 and cPIM-1 TFCs fabricated from THF solutions. 33

References 35

S1. Gas permeation performance of self-standing PIM-1 films.

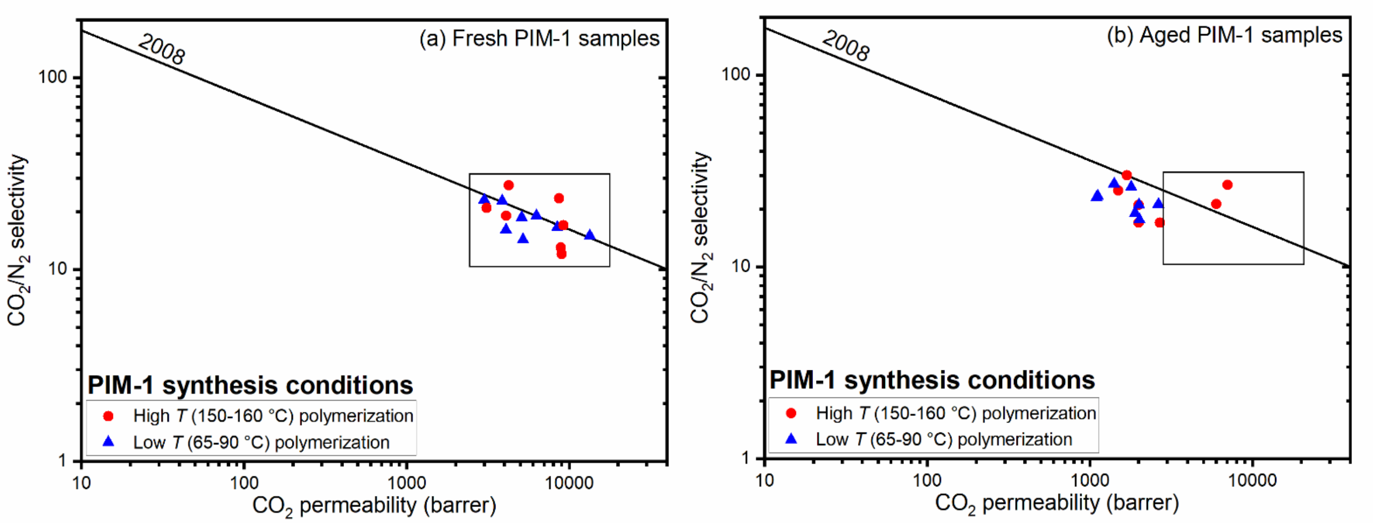

Figure S1. Selection of ideal CO_2/N_2 separation performances of (a) fresh and (b) aged self-standing PIM-1 film membranes reported in the literature.[1-14]

Note: PIM-1 polymers used to prepare the respective membranes are grouped into two categories, high *T* or low *T*, based on the polymerization temperature used in their synthesis.

Table S1. Reported ideal CO_2/N_2 performance of self-standing PIM-1 membranes (fresh and aged) prepared from different polymers synthesized under the outlined polymerization conditions. [1-14]

PIM-1 polymer ref.	Polymerization details				Self-standing film membrane performance					
	Temp. / °C	Time / h	Solvent	Polymer M_w / kg mol^{-1}	Age / day	$P(CO_2)$ / barrer	CO_2 / N_2	Age / day	$P(CO_2)$ / barrer	CO_2 / N_2
[1]	60	48	DMF	80-100	1	5200	14.3	450	1800	26
[2]	75	60	DMF	110	1	4087	16	92	1900	19
[3]	70	72	DMF	266	1	8440	16.5	180	2020	17.6
[4]	155	4	NMP + toluene	-	1	4250	27.5	100	1700	30
[5]	160	3.5	NMP + toluene	-	1	4100	19	100	1500	25
[6]	90	8	DMF	217	1	5105	18.7	180	2640	21.1
[7]	160	3	DMF + mesitylene	242	15	8700	23.5	60	7047	26.8
[8]	65	72	DMF	174	1	3000	23	70	1410	27
[9]	65	72	DMF	-	1	13400	15	410	2010	21
[10]	85	24	DMF	-	1	3873	22.7	60	1117	23.4
[11]	160	0.5	DMAc + toluene	116	1	9000	12	145	2000	17
	160	0.67		143	1	8900	13	145	2700	17
[12]	162	0.75	DMAc	-		9200	17	100	6000	21.2
[13]	150	-	DMAc	74.8	1	3104	20.9	30	2004	21
[14]	65	72	DMF	-	1	6300	19	120 (80 °C)	1100	23

S2. Gas permeation performance of PIM-1 thin film composites (TFCs), PIM-1 based thin film nanocomposites (TFNs) and modified PIM-1 TFCs fabricated by different methods.

Table S2. Reported ideal CO_2/N_2 performance of PIM-1 TFC membranes (fresh and aged) prepared via dip-, kiss-, spin- coating or other methods (presented in **Figures 1(a) & 1(b)**).

Ref.	PIM-1 fabrication details					TFC membrane performance					
	Coating method	Solvent	Conc./ wt%	Support	Active layer /μm	Age / day	$K(CO_2)$ / GPU	CO_2/N_2	Age / day	$K(CO_2)$ / GPU	CO_2/N_2
15	Dip	$CHCl_3$	1	PAN (25 nm)	0.75	1	7147	28.1	-	-	-
2	Spin	THF	1	AAO (20 nm)	0.2	1	16917	15	-	-	-
			5		2.5	1	2403	15.6	-	-	-
16	Dip	$CHCl_3$	1	PAN (22nm)	0.2	1	4100	21.3	-	-	-
17	Dip	$CHCl_3$	2	PAN (31nm)	2	1	3300	14.2	90	380	24
18	Dip	DCM	1	PDMS@PAN	1.45	1	403	21.3	-	-	-
19	Dip	$CHCl_3$	2	uv x-linked PEG	2	1	1874	12	-	-	-
20	Kiss	$CHCl_3$/ C_2HCl_3 (1 : 1)	0.5	PDMS@PAN	0.29	1	8010	35.8	98	208	34.6
					0.34	1	4930	38.8	94	297	37.1
					0.42	1	3010	55.7	95	281	56.2
21	Dip	$CHCl_3$	2	PAN	2.7	1	416	28.8	-	-	-
22	Kiss	$CHCl_3$	2	PAN	2.16	1	5079	12.4	28	395	6.5
					1.86	1	5985	19.9	28	493	14.1
3	Spin	$CHCl_3$	1	PDMS@MOF @PAN	0.65	1	4340	18.9	14	460	16.1
23	Spin	$CHCl_3$	1	PDMS@MOF @PAN	0.65	1	4320	19	56	600	31
24	Dip	THF	1	PAN (22nm)	0.4	1	3400	21	-	-	-
25	Spray	$CHCl_3$	0.5	PAN	2.75	1	159	12	-	-	-
26	Kiss	$CHCl_3$	2	PAN	1.92	1	4678	11.2	28	134	3.6
					1.33	1	2903	5.8	28	343	1.8
					2.7	1	4642	13.3	28	671	27.7
27	Bar	THF	4	PAN	3.3	1	2000	14	14	100	25

28	Kiss	10% THF in $CHCl_3$	4.5	PAN	2	1	4599	21	28	1331	15.4
11	Kiss	THF	3.3	PAN	3.72	1	6200	14	60	930	23
					3.62	1	3100	19	60	1100	20
29	Kiss	THF	3.3	PAN	-	1	2100	21	28	1400	19
30	Spin			PAN	2.6	1	2100	16.2	14	130	26
31	Bar	$CHCl_3$	-	PDMS@PES	-	1	755	23.6	-	-	-
32	Kiss	$CHCl_3$	2	PAN	2.5	1	3516	12.4	28	644	16
33	Spin	$CHCl_3$	0.5-5.0	AAO	0.183	1	8600	17	7	950	42
					1.46	1	2800	17	7	950	33

Table S3. Reported ideal CO_2/N_2 performance of PIM-1 TFN membranes or modified PIM-1 TFC membranes (fresh and aged) prepared via dip-, kiss-, spin- coating or other methods (presented in **Figures 1(c) & 1(d)**).

Ref.	TFN fabrication details				TFN membrane performance					
	Coating method	Solvent	PIM-1 blended TFNs or modified PIM-1 TFCs	Active layer / µm	Age / day	$K(CO_2)$ / GPU	CO_2/N_2	Age / day	$K(CO_2)$ / GPU	CO_2/N_2
15	Dip	$CHCl_3$	2 wt % f-MWCNTs	0.75	1	9453	32.9	-	-	-
2	Spin	THF	5 wt % OAPS	0.2	1	13,585	19.2	-	-	-
			5 wt % OAPS	2.5	1	2138	19.4	-	-	-
16	Dip	$CHCl_3$	33wt % f-GO	0.2	1	3000	21.1			
17	Dip	$CHCl_3$	60wt% C-HCP	2	1	27,500	7	90	9300	11
18	Dip	DCM	modified, PIM-CD	1.32	1	483	22.5			
19	Dip	$CHCl_3$	modified, Al^{3+} x-linked cPIM-1	2	1	1058	23.5			
3	Spin	$CHCl_3$	modified, PIM-C1	0.65	1	6400	29.1	14	220	5.9
23	Spin	$CHCl_3$	10wt% MOF-74-Ni	0.65	1	5018	31	56	1200	33
			10wt% NH_2-UiO-66	0.65	1	7460	26	56	900	26
24	Dip	THF	10wt% Zn2$(bim)_4$		1	5000	15.4	-	-	-
25	Spray	$CHCl_3$	18 wt% HKUST-1	2.75	1	696	6.4	-	-	-
26	Kiss	$CHCl_3$	9 wt% CN-PIM-1a	1.92	1	5410	12.6	28	287	18.1
			18 wt% CN-PIM-1a	1.92	1	5910	16.6	130	35	10.7
			5 wt% CN-PIM-1b	1.92	1	4708	15.2	130	76	5.5
			10 wt% CN-PIM-1b	1.92	1	4356	13.5	130	41	11
27	Bar	THF	20 wt% polyMOF		1	4800	22	14	900	32
28	Kiss	10% THF in $CHCl_3$	8.5wt% C-UiO-66-NH_2/cPIM-1	2	1	2763	29.4	63	2504	37.2
11	Kiss	THF	modified, D-cPIM-1 70 %	0.63	1	7700	56	60	3700	40
			modified B-cPIM-1 73 &81 %	1.9	1	3200	64	60	5000	110

31	Bar	$CHCl_3$	8wt% UiO-66-$(CF_3)_2$	-	1	1255	23.6	30*	1000*	44*
32	Kiss	THF	modified, B-cPIM-1	1.1	1	4671	65.6	28	1483	27.8
			B-cPIM-1 cross-linked with 3 mol % Co-MOF-74	2.2	1	4297	84.7	28	717	37.3

S3. PIM syntheses and nomenclature for polymers used in TFC and TFN membranes.

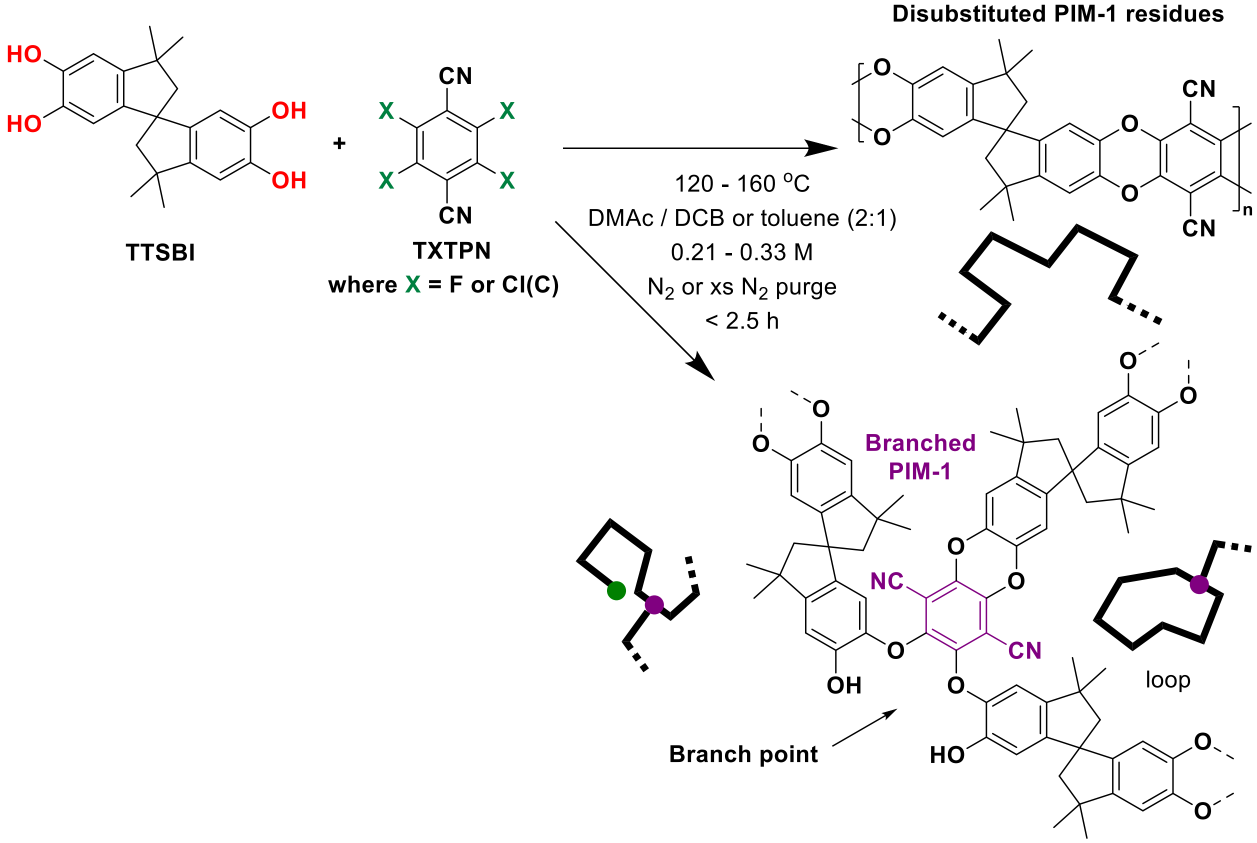

Scheme S1. PIM-1 polymerization.

Full details on the PIM-1 synthesis and characterization obtained for each of the polymers presented in **Table 1** are provided in the original publications, which are referenced next to the assigned polymer name in the main paper. The referencing system for the polymers in the main paper is also carried across and used for simplicity in polymer names presented in **Tables S6-9** and **Figures S7-12**. A prefix description to PIM-1 is assigned if a dominant soluble polymer topology can be assigned to a particular sample: D-PIM-1 refers to a predominantly di-substituted polymeric sample, whilst B-PIM-1 refers to a branched polymeric sample (> 10 %) and T-PIM-1 refers to a particular sample with optimised topology which included small loops. Information in brackets relates to the respective tetrahalo monomer used (C = TCTPN or F = TFTPN) with TTSBI in synthesis (3-fold molar excess of K_2CO_3 used in all cases) and the set point temperature for the respective polymerizations (**Table 1**). The reaction temperature is quoted in the case of the very large-scale, longer duration polymerization (150 mmol), defined as B-PIM-1(C, 120 °C)[33]. The base was also only added once internal temperature reached 60 °C, whereas in all other polymerizations the base was present in reaction mixtures at commencement of heating up from RT to

the set temperature. The PIM-1 synthesis conditions used to obtain the polymers are presented grouped in terms of standard solvent concentrations in **S3.1.** (**Table S4**) and under more dilute conditions in **S3.2.** (**Table S5**). The performances of TFCs fabricated from each of these PIM-1 polymers are presented in **Figure 2** (**Table S6**). Aging comparisons relating to discussions in the main text are sequentially presented in **Figures S7-S9**.

It should be noted that aspects of the PIM-1 synthesis and characterisation details for PIM-1(F, 160 °C)[18] are incorrectly stated in the literature.[28] In addition to the 20 % extra of solvent mixture present at the start, extra batches of DMAc (20 ml) and toluene (10 ml) were added after 9 and 13 min of the overall 30 min reaction (**Table S5**). This PIM-1 polymer exhibited M_w = 152.5 kg mol^{-1} and dispersity, $Đ_M$ = 2.1.

Full details on the PIM synthesis and characterization obtained for the three colloidal network (CN) rich PIM polymers, defined as CN-PIM-1a[16], CN-PIM-1b[16] and CN-Cardo-PIM-1[34] in **Table 1**, which were prepared as TFNs in blending with PIM-1 polymers, are provided in the original publications, with reference numbers next to polymer name here again carried across from the main paper. The performance of TFNs fabricated from each of these colloidal network PIM polymers blended with a base PIM-1 polymer are presented, alongside base polymer TFC performance, in **Figure 4** (**Table S7**). Aging comparisons relating to discussions in the main text are sequentially presented in **Figures S10-12**.

Full details on other PIM-1 polymers synthesized as part of blending studies with other fillers for TFNs are provided in the original publications. The performance of TFNs fabricated from these other fillers blended with a base PIM-1 polymer are presented, alongside base polymer TFC performance, in **Figures 5-7** (**Table S8**). Full details of other PIM-1 samples synthesized and then modified to cPIM-1 for TFCs fabricated from THF solutions are also provided in the original publications. The performance of TFCs of both base PIM-1 and hydrolysed cPIM-1 polymers are presented in **Figures 8 & 9** (**Table S9**).

S3.1. PIM-1 polymerizations (0.33 M).

Equimolar amounts of the monomers, TCTPN(C) or TFTPN(F) (***A*** mmol) and TTSBI (***A*** mmol), were placed along with potassium carbonate (***3A*** mmol) into a three-necked, round-bottom flask. The solvent mixture of DMAc (2***B*** mL) and DCB (***B*** mL) was then added. The flask was equipped with a nitrogen inlet, a coil condenser, and a mechanical stirrer (complete with digital rpm and torque reading display) which was used to mix the reaction mixture. A strong positive pressure of nitrogen ($\boldsymbol{N_2}$ or ***xs*** $\boldsymbol{N_2}$) was maintained over the reaction mixture. Heat was supplied via a hotplate equipped with a DrySyn aluminium heating block, with the flask inserted together with a temperature probe. The reaction mixture was heated from room temperature to set temperature, ***T*** °C, initially stirred at 200 rpm, with the stirring rate

increased gradually as appropriate and torque readings recorded at regular intervals. The viscosity of the reaction mixture facilitated increasing the stirrer rate to its maximum setting of 500 rpm within completion of polymerization. After ***X*** min, the hot viscous reaction mixture was quenched into excess methanol, precipitating as a yellow PIM-1 polymer.

Table S4. PIM-1 synthesis conditions for 0.33 M polymerizations presented in **Table 1**.

Ref.	Polymer	PIM-1 synthesis conditions				
		Scale, ***A*** / mmol	Solvent mixture, ***3B*** / ml	Purging conditions	Set temperature, ***T*** / °C	Reaction time, ***X*** / min
22	PIM-1(C,160 °C)[11]	10	30	N_2	160	33
22	PIM-1(C,120 °C)[11]	10	30	N_2	120	81
26	D-PIM-1(C,140 °C)[16]	10	30	xs N_2	140	53
26	D-PIM-1(C,120 °C)[16]	10	30	xs N_2	120	75
34	B-PIM-1(C, 120 °C)[33*]	150	450	N_2	120	150
35	B-PIM-1(F,160 °C)[34]	30	90	N_2	160	38

*K_2CO_3 only added to reaction mixture once internal temperature reached 60 °C.

S3.2. Diluted PIM-1 polymerizations (0.27/0.21 M).

Equimolar amounts of the monomers, TCTPN (C) or TFTPN (F) (***A*** mmol) and TTSBI (***A*** mmol), were placed along with potassium carbonate (***3A*** mmol) into a three-necked, round-bottom flask. The solvent mixture of DMAc (***2.4B*** mL) and DCB or toluene (***1.2B*** mL) was then added. The flask was equipped with a nitrogen inlet, a coil condenser, and a mechanical stirrer (complete with digital rpm and torque reading display) which was used to mix the reaction mixture. A strong positive pressure of nitrogen ($\boldsymbol{N_2}$) was maintained over the reaction mixture. Heat was supplied via a hotplate equipped with a DrySyn aluminium heating block, with the flask inserted together with a temperature probe. The reaction mixture was heated from room temperature to set temperature, 160 °C, initially stirred at 200 rpm, with the stirring rate increased gradually as appropriate and torque readings recorded at regular intervals. In some cases, the reaction mixtures were further diluted, after ***C*** min, by adding an extra batch of the DMAc (***0.4B*** mL) and toluene (***0.2B*** mL) solvent mixture to the flask. This solvent batch addition (***0.6B***) was repeated after ***D*** min. The viscosity of the reaction mixture facilitated increasing the stirrer rate to its maximum setting

of 500 rpm within completion of polymerization. After ***X*** min, the hot viscous reaction mixture was quenched into excess methanol, precipitating as a yellow PIM-1 polymer.

Table S5. PIM-1 synthesis conditions for 0.27 (/0.21) M polymerizations presented in **Table 1**.

Ref.	Polymer	PIM-1 synthesis conditions				
		Scale, ***A*** / mmol	Solvent mixture, ***3.6B*** / ml	Solvent addition times (+0.6B), ***C, D*** / min	Total solvent mixture at end / ml	Reaction time, ***X*** / min
26	T-PIM-1(C, 160 °C)[16]	50	180	-	180	40
28	PIM-1(F, 160 °C)[18]	50	180	9, 13	240	30
32	PIM-1(F, 160 °C)[23]	50	180	15, 22	240	46

S4. Estimation of loops in average PIM-1 polymer chain.

An estimation of number of loops (l) present in an average PIM-1 polymer chain is based around **equation S1**, which has been previously applied to the characterization of T-PIM-1(C, 160 °C).[26] End group NMR analysis, or more commonly elemental analysis of chlorine content in the polymer compared against the number average molar mass of the polymer determined by SEC analysis, allows estimation of number of chloro ends (e) remaining on an average PIM-1 polymer chain. The number of branching (b) units present on an average PIM-1 polymer chain can be determined by measuring the extent of the diminishment of the peak associated with the fully conjugated PIM-1 residue structure in UV-vis analysis of the polymer in tetrahydrofuran (THF) solution,[26] or from integral area analysis of the peaks associated with branching present in the aromatic region of proton NMR spectrum.[36] Rearranging the equation based on estimations for e and b allow indirect determination of the number of loops (l) present in an average PIM-1 polymer chain.

$$e = 2 + b - 2l \qquad \textbf{Equation S1}$$

Two examples of where this estimation has been applied to determine the topology of PIM-1 polymers synthesized from TCTPN (C) monomer are explained in full in the next sections. The level of branching can generally be readily measured from NMR analysis, but often the polymer samples don't have enough end groups to obtain a measurable amount of chlorine (although it can also be inferred sometimes from a lower than expected carbon content). Heavier chlorine, rather than fluorine, atoms on polymer ends also provide a better opportunity to measure residual halogen content on these polymers, and additionally chlorine analysis is readily available as part of most university analytical services.

S4.1. Topology estimation for T-PIM-1(C, 160 °C)[16] polymer.

Elemental analysis of T-PIM-1 polymer did not register a measurable amount of chlorine (Cl content < 0.30 wt %). Considering the detection limit for chlorine, this was qualified as < 2.5 Cl atoms potentially present on an average polymer chain (e = 0 or 1). NMR analysis of the aromatic proton region showed branching peaks but no evidence of chloro ended polymeric chains. This suggested that the branched structures are part of loop structures. This sample contained a low level of colloidal network material (6.6 wt %). This suggested that the reaction conditions chosen, under nitrogen, favoured loop formation over secondary reactions which form four-way linkages, which can also remove excess chlorines previously left on the ends of branches. The overall intermediate molar mass obtained in the reaction (M_w = 62,400) would also point to the limitation of branch points not involved in loops. UV-vis analysis of T-PIM-1 in solution indicated the presence of one branch (defect) per every 16.4 spiro (***S***) residues. This equated to 4 branch points per 67 PIM-1 residues (M_n = 30,500) overall. A polymeric structure with on average four branch points (b = 4) but no ends (e = 0) must contain three loops (l = 3).

S4.2. Topology estimation for B-PIM-1(C, 120 °C)[33] polymer.

Elemental analysis of B-PIM-1(C, 120 °C) polymer indicated the presence of a considerable amount of chlorine (Cl content = 1.42 wt %). This was qualified, based on the average molar mass of polymer (M_n = 49,400) to indicate as many as 20 Cl atoms remaining on an average polymer chain (e = 10). Unusually for PIM-1 analysis, the C/N elemental ratio for this polymer indicated that there was an excess of two spiro (***S***) residues in the overall polymeric structures. These two spiro (***S***) residues have the capacity to consume a further four chloro (***C***) residue ends, either to chain extend the polymer or form part of a loop or ring structure. We will assume that these extra spiro (***S***) residues are extending the polymer chain and are not involved in loop or ring structures, as we have no means to prove that they are. This means effectively that the theoretical number of end groups not involved in loops is 14, e = 14 (basis of original equation was that there are equal amounts of both residues in an average polymer chain). Integral area analysis of the very significant peaks associated with branching in the aromatic region of the proton NMR spectrum indicated that this polymer was 22 % branched. This equates to 24 branch points per 107 PIM-1 residues (M_n = 49,400) overall. A polymeric structure with on average twenty-four branch points (b = 24) but only 14 ends (e = 14) must contain six loops (l = 6). Give the earlier assumption made, it is perhaps best to state that there are at least 6 loops present in an average polymer chain of this polymer.

S5. DSC analysis of PIM-1 polymers.

DSC analysis was completed on some representative PIM-1 samples: predominantly di-substituted PIM-1 (**D-PIM-1**, M_w = 106.2 kg mol^{-1}, $Đ_M$ = 1.8, branching = 2.4 %, colloidal network content < 2 %), heavily branched PIM-1 polymer (**B-PIM-1**, M_w = 69.9 kg mol^{-1}, $Đ_M$ = 2.0, branching = 11 %, colloidal network content = 10 %), colloidal network rich **CN-Cardo-PIM-1** (M_w = 47.6 kg mol^{-1}, $Đ_M$ = 22, branching n/a, colloidal network content = 70 %) and polymer film prepared from **B-PIM-1(F, 160 °C)[34] blended with 3.5 wt % CN-Cardo-PIM-1**. A Perkin Elmer DSC8000 instrument, which uses the power compensation principle, and Perkin Elmer Pyris software were utilized. Polymer powder and blended film samples (2-10 mg) were treated to repeated heating and cooling cycles between 30 °C to 450 °C at 10 °C min^{-1}. The enlarged high temperature regions (440-445 °C) of cooling cycles of the DSC traces obtained for **D-PIM-1**, **B-PIM-1 and B-PIM-1(F, 160 °C)[34] + 3.5 wt % CN-Cardo-PIM-1** are presented in **Figure 3**. The four complete DSC traces obtained for the polymer samples are presented in **Figures S2-S5**.

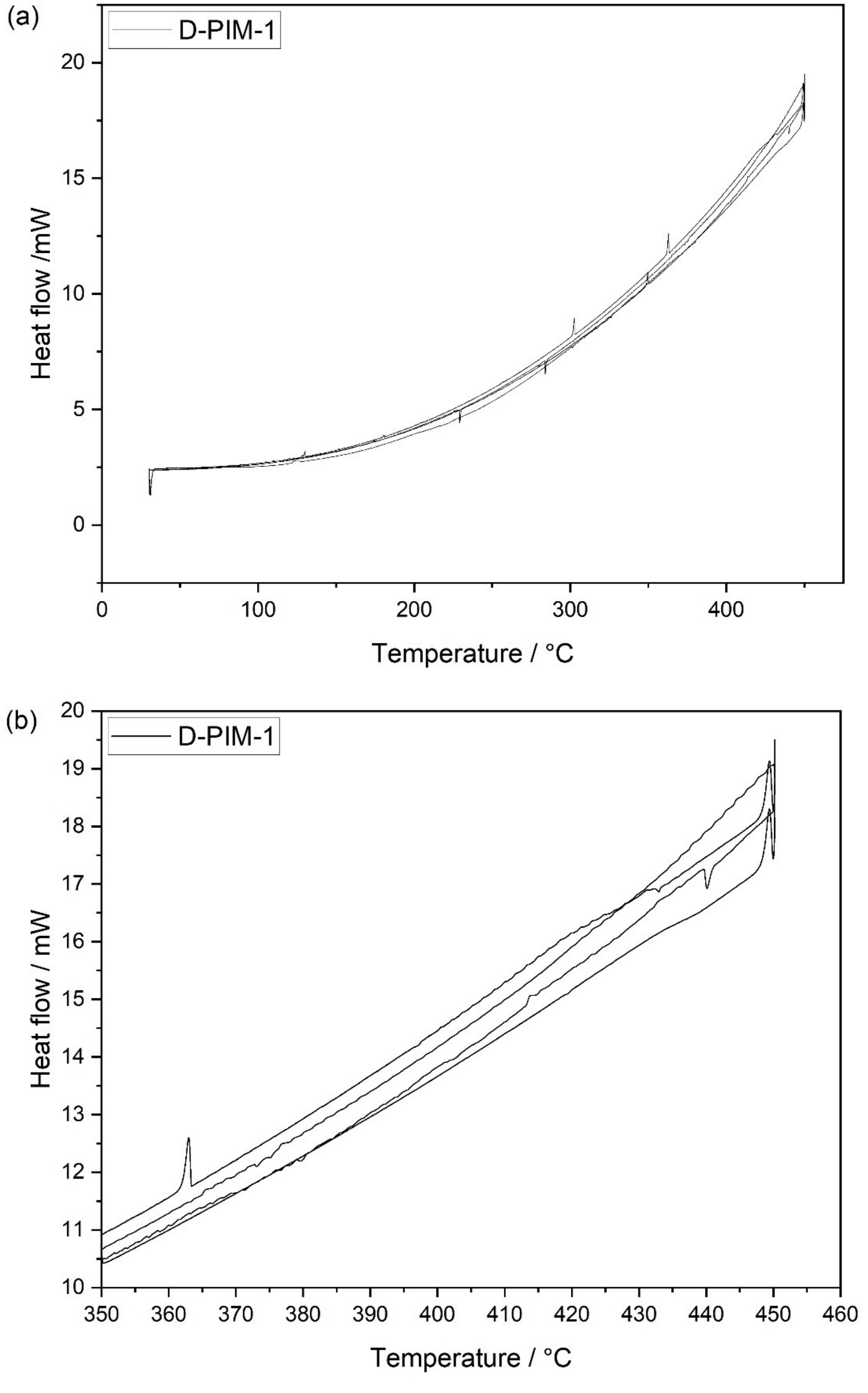

Figure S2. (a) Full DSC trace and (b) expanded region of D-PIM-1 polymer.

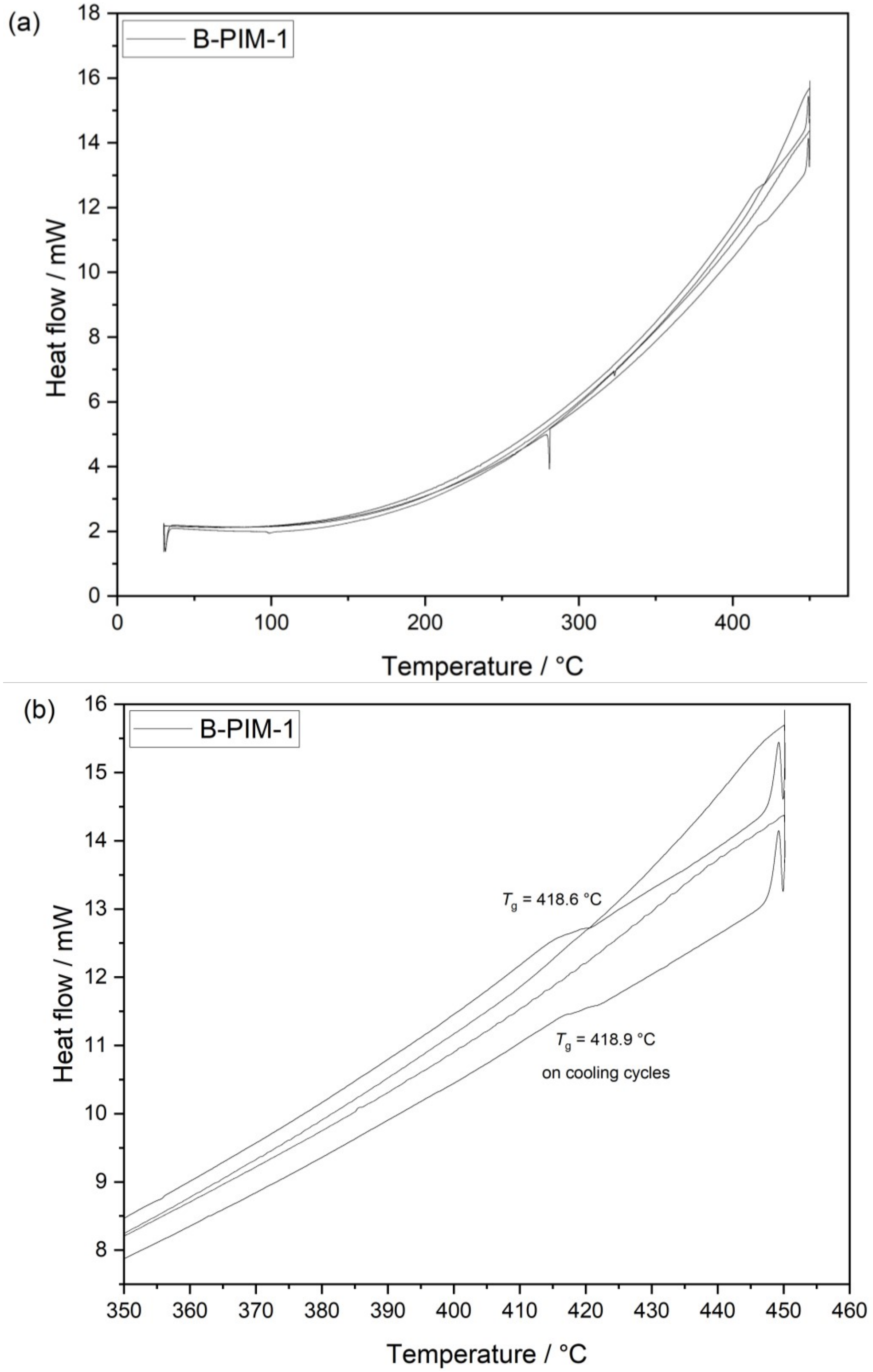

Figure S3. (a) Full DSC trace and (b) expanded in region of defined glass transition of B-PIM-1 polymer. T_g = 419 °C.

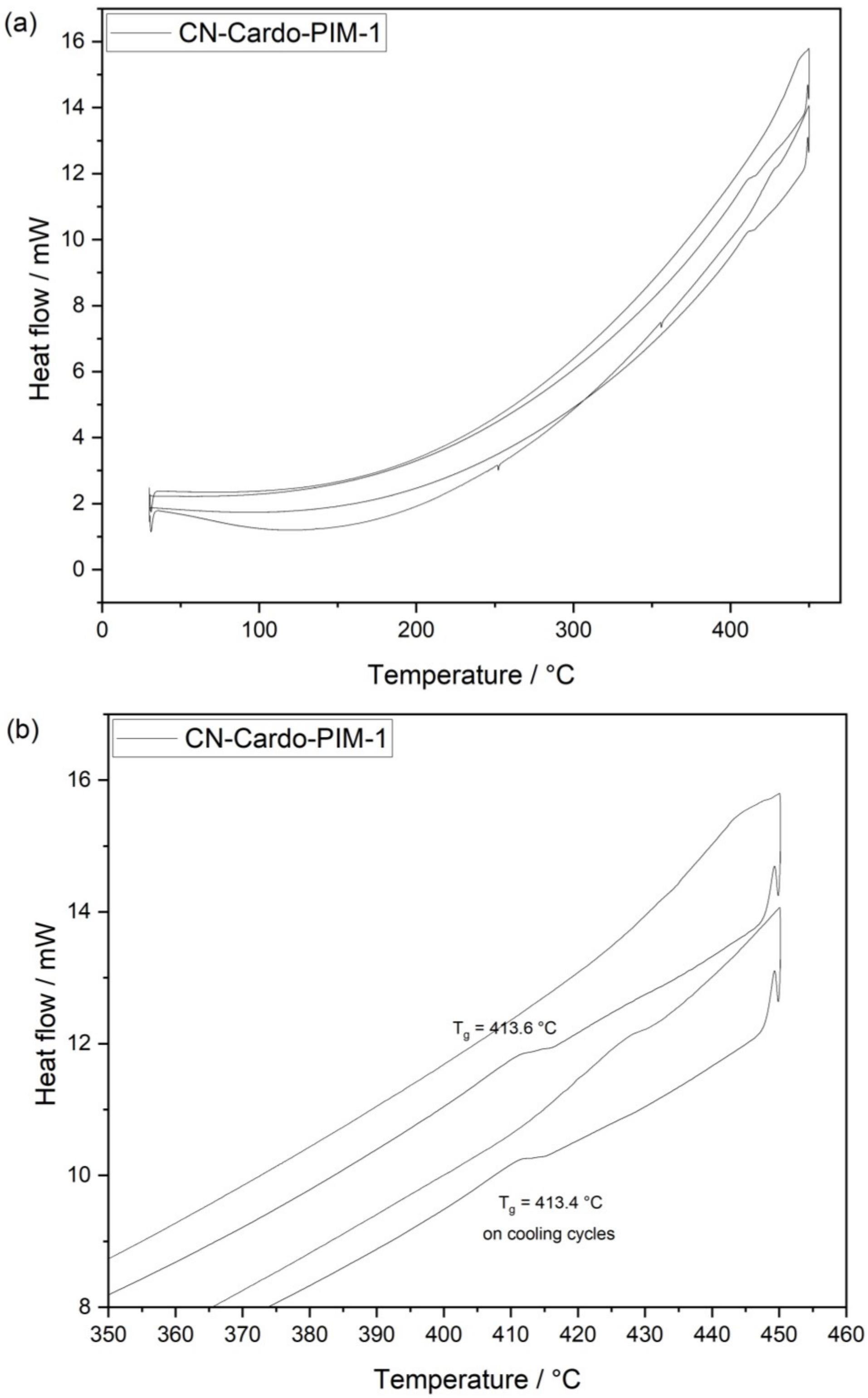

Figure S4. (a) Full DSC trace and (b) expanded in region of defined glass transition of CN-Cardo-PIM-1 polymer. T_g = 414 °C.

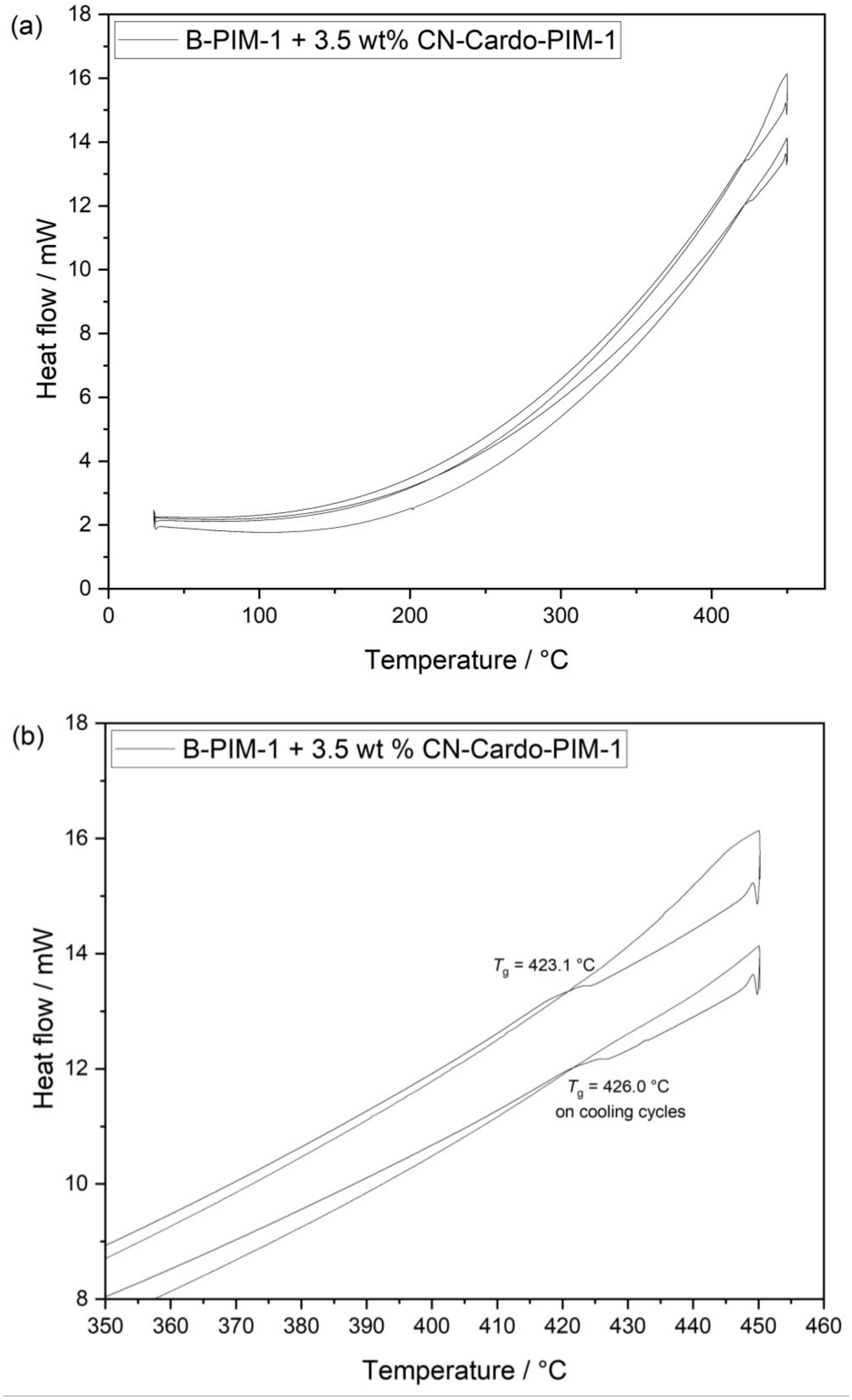

Figure S5. (a) Full DSC trace and (b) expanded in region of defined glass transition of self-standing film prepared from B-PIM-1 blended with 3.5 wt % CN-Cardo-PIM-1 polymer. T_g = 423-426 °C.

S6. Fabrication of TFCs and TFNs via kiss-coating procedure.

S6.1. PIM solutions used in TFC/TFN fabrication.

TFCs or TFNs were fabricated from 3 % w/v solutions of the PIM-1 polymer or PIM-1 blend in chloroform (typically 0.3 g in 10 ml $CHCl_3$), unless stated otherwise in the main text, Table or Figure caption. Polymer blends which contain network rich polymeric material[26, 35] are defined in **Figure 4** (**Table S7**) in relation to the overall actual content of colloidal network present. For example, the best performing TFN blend reported was for B-PIM-1(F, 160 °C)[34] blended with 5 % CN-Cardo-PIM-1, which is comprised of 70 % colloidal network, meaning overall network content in blend was 3.5 %.[35] The conditions under which other TFCs or TFNs of PIM-1 and other fillers were fabricated are provided in the original publications (**Figures 5-7**, **Table S8**). Higher solution concentrations (up to 6 % w/v) were often required for kiss-coating of cPIM-1 from THF owing to lower solution viscosity (**Figures 8 & 9, Table S9**).

S6.2. Thin film composite membrane preparation (kiss-coating technique).

Thin film composite (TFC) and thin film nanocomposite (TFN) membranes which have been fabricated in Manchester via the kiss-coating technique have employed three different sized roller-coaters. At least three samples of similar ultrafiltration (UF) support, originating from a company formerly trading as Sepro Membranes Inc (California, USA), have provided the backing support materials used in our work since 2018. Samples of the most widely used product, defined in Tables as PA350, was kindly supplied by Prof. Ingo Pinnau at different times over recent years. The PA350 UF support exhibits a narrow pore size distribution, with a pore size ~ 31 nm.[17] Other products used from Sepro, include PS35, which was obtained indirectly by Ph.D. students from Saudi Aramco.

For the largest roller coater, PAN ultrafiltration (UF) support was cut into a rectangular sheet with dimensions of 4.5 cm × 10 cm. The support was then attached to the roller wheel with edges sealed with aluminum tape to prevent any solution soaking to the bottom of the support. The roller-coater was connected to a programmable DC power supply motor (RS-3005P, RS PRO, UK) with voltage of 15 V and current reading around 0.14 A. Polymer solution was prepared as 3-6 % w/v in either chloroform or THF and was poured into a steel tray below the coater with height adjusted by placing glass slides underneath. After coating, the sheets were peeled off from the roller and placed in a nitrogen cabinet at room temperature overnight before any testing. The overall set up for the largest roller coater is presented in **Figure S6**.

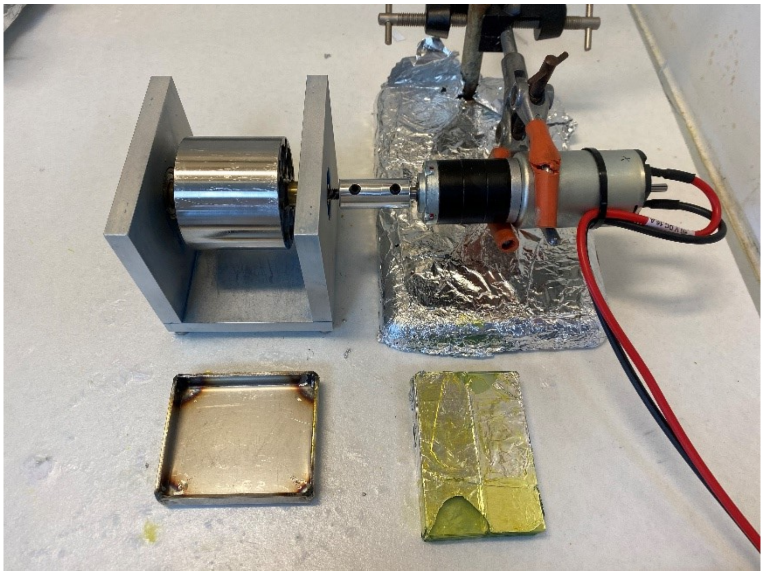

Figure S6. Thin film kiss-coating setup with a steel roller coater connected to a motor, steel tray for containing coating solution, and glass plates for supporting the tray to control the contact between PAN support and solution.

S7. Gas permeation tests.

Gas permeance tests were performed at room temperature by the standard variable volume method,[37] and N_2, CH_4 and CO_2 pure gases were used in that sequence. Upstream gauge pressure was maintained at 35 psi (2.41 bar) with downstream at atmospheric pressure. PIM-1 TFC membranes were cut into circular coupons to fit the testing rig with active permeation area of 2.84 cm^2. Before collecting any data, the membranes were pre-conditioned under the testing pressure for 5 min for each gas. Then the time for a specific volume of gas to permeate through the membrane was recorded. Membrane permeance was calculated based on **Equation S2**:

$$K = \frac{Q}{tA(p_1 - p_2)} \times 10^6 \qquad \textbf{Equation S2}$$

In this equation, K is the gas permeance (GPU, 1 GPU = 10^{-6} cm^3 [STP] cm^{-2} s^{-1} $cmHg^{-1}$ = 3.348×10^{-10} mol m^{-2} s^{-1} Pa^{-1}), t is the permeation time (s), Q is the volume of gas that permeates through the membrane during the permeation time (cm^3, corrected to STP [0 °C, 1 atm]), A is the active permeation area (cm^2), and p_1 and p_2 are the pressure in the membrane feed side and permeate side (cmHg), respectively.

The membrane ideal gas selectivity, α, was calculated as the ratio of gas permeances by **Equation S3**:

$$\alpha_{CO_2/x} = \frac{K_{CO_2}}{K_x} \qquad \textbf{Equation S3}$$

where *x* is either N_2 or CH_4. Sufficient TFC membranes were typically fabricated to ensure that it was not necessary to re-test an individual TFC for a second time at a later aging time interval. TFC performance was tracked to measure physical aging across at least a period of 28 days and sometimes up to 1 year. At least two membranes of each sample were tested for reproducibility, and the average with standard deviation is reported.

S8. Thin film composite (TFC) performance of topologically distinct PIM-1 polymers.

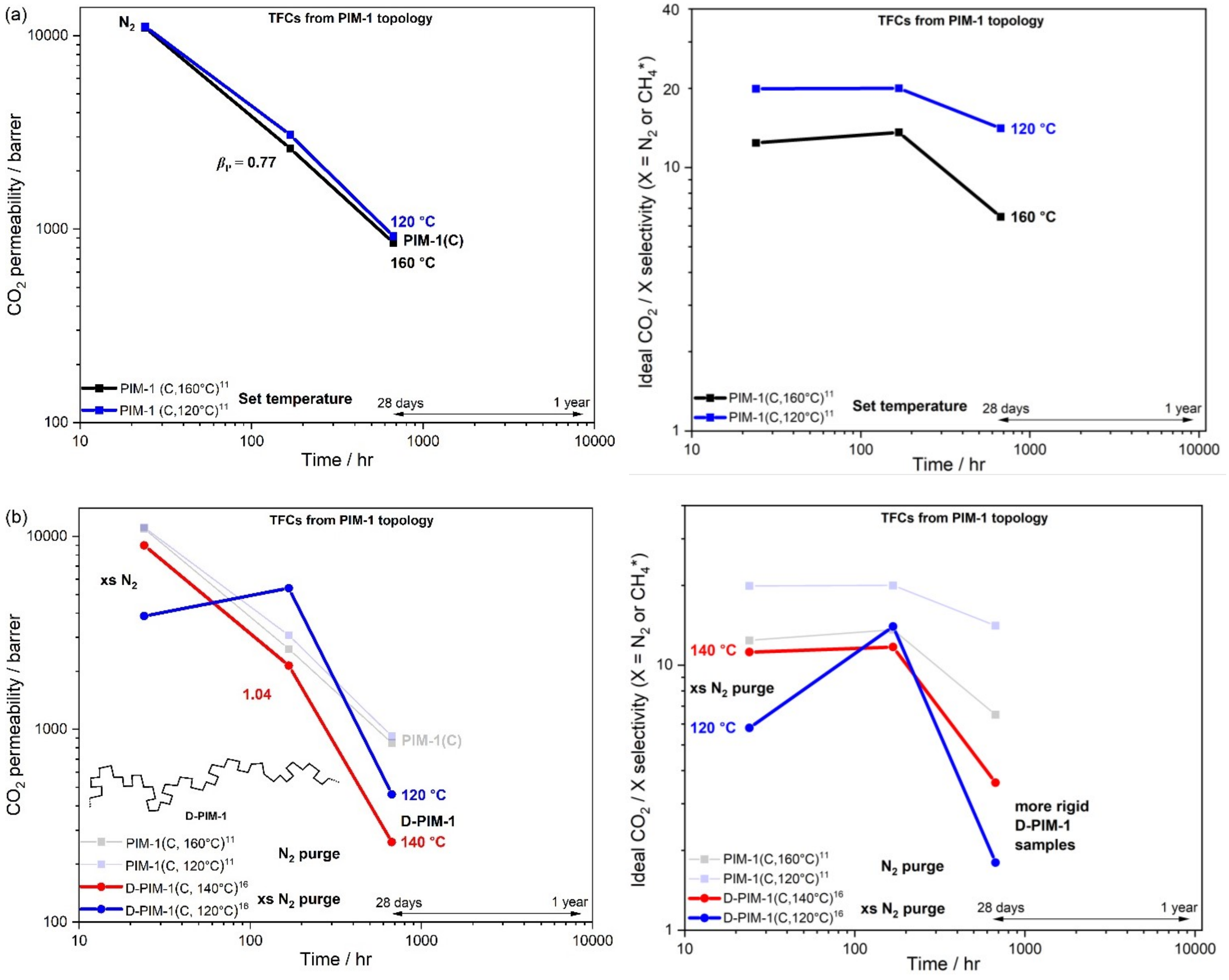

Figure S7.(a,b) Comparisons discussed within TFC aging data fabricated from different PIM-1 polymers presented in **Figure 2**.

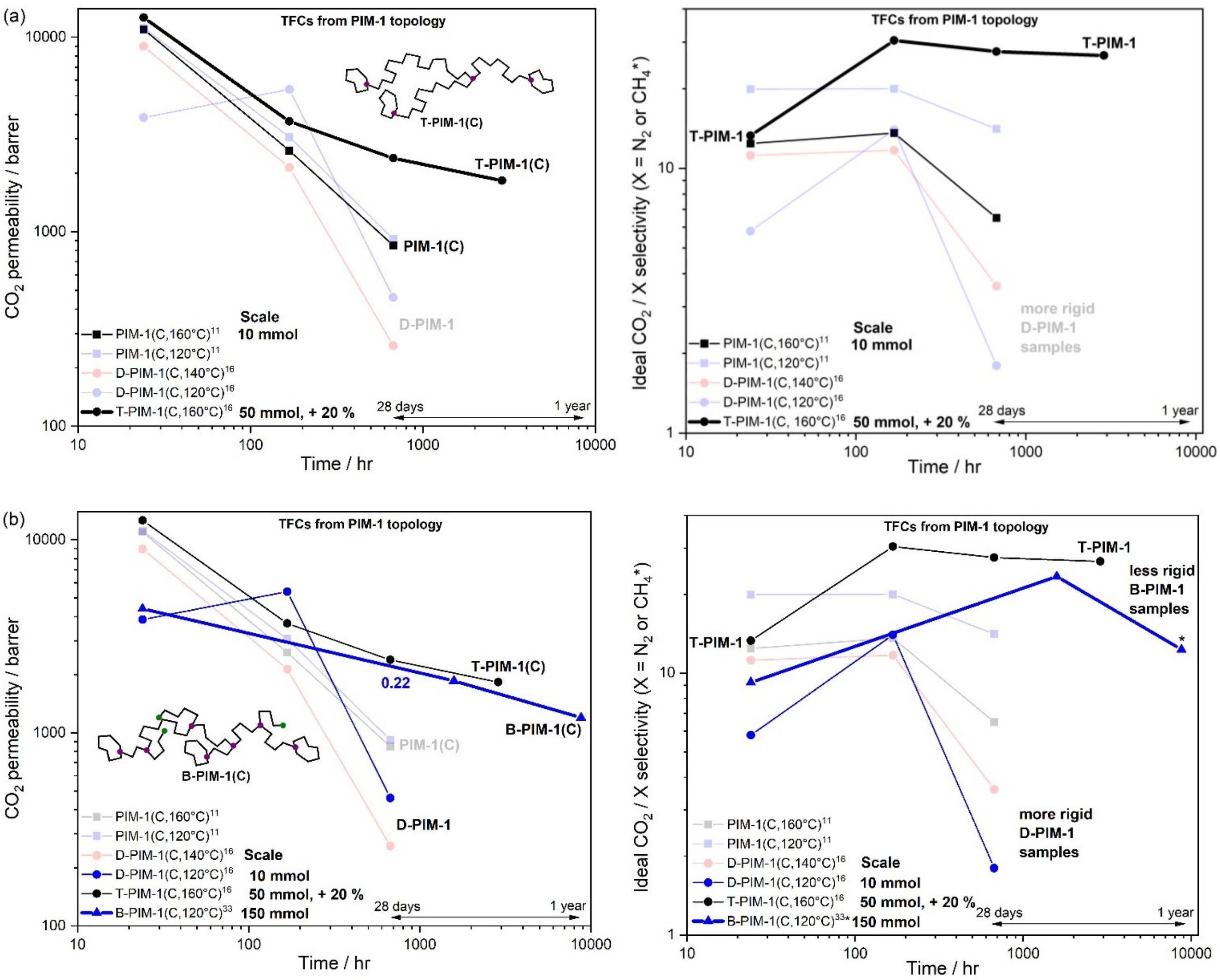

Figure S8.(a,b) Comparisons discussed within TFC aging data fabricated from different PIM-1 polymers presented in **Figure 2**.

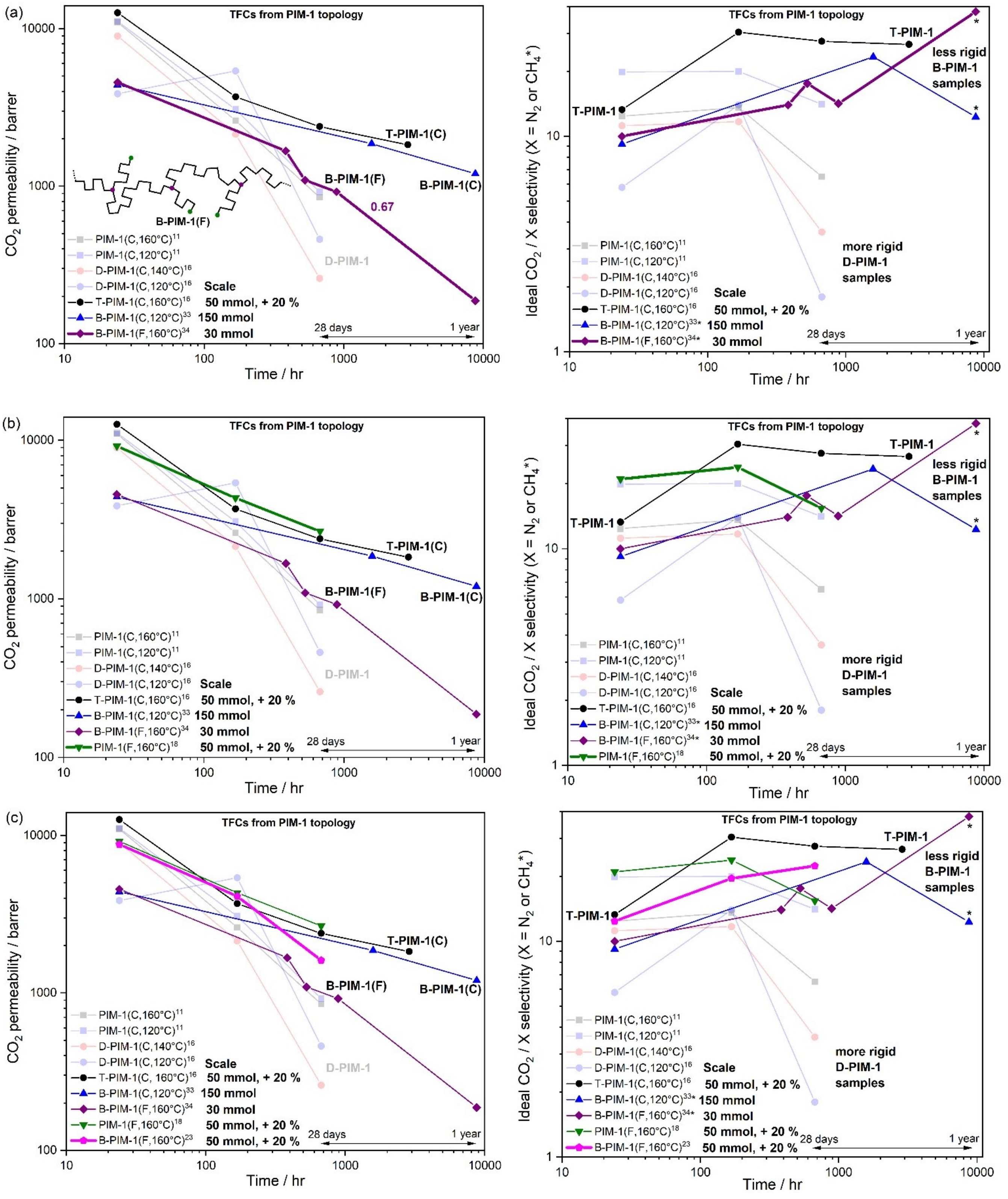

Figure S9.(a-c) Comparisons discussed within TFC aging data fabricated from different PIM-1 polymers presented in **Figure 2**.

S9. Thin film composite (TFC) and thin film nanocomposite (TFN) performance of PIM-1 polymers blended with network rich PIM polymers.

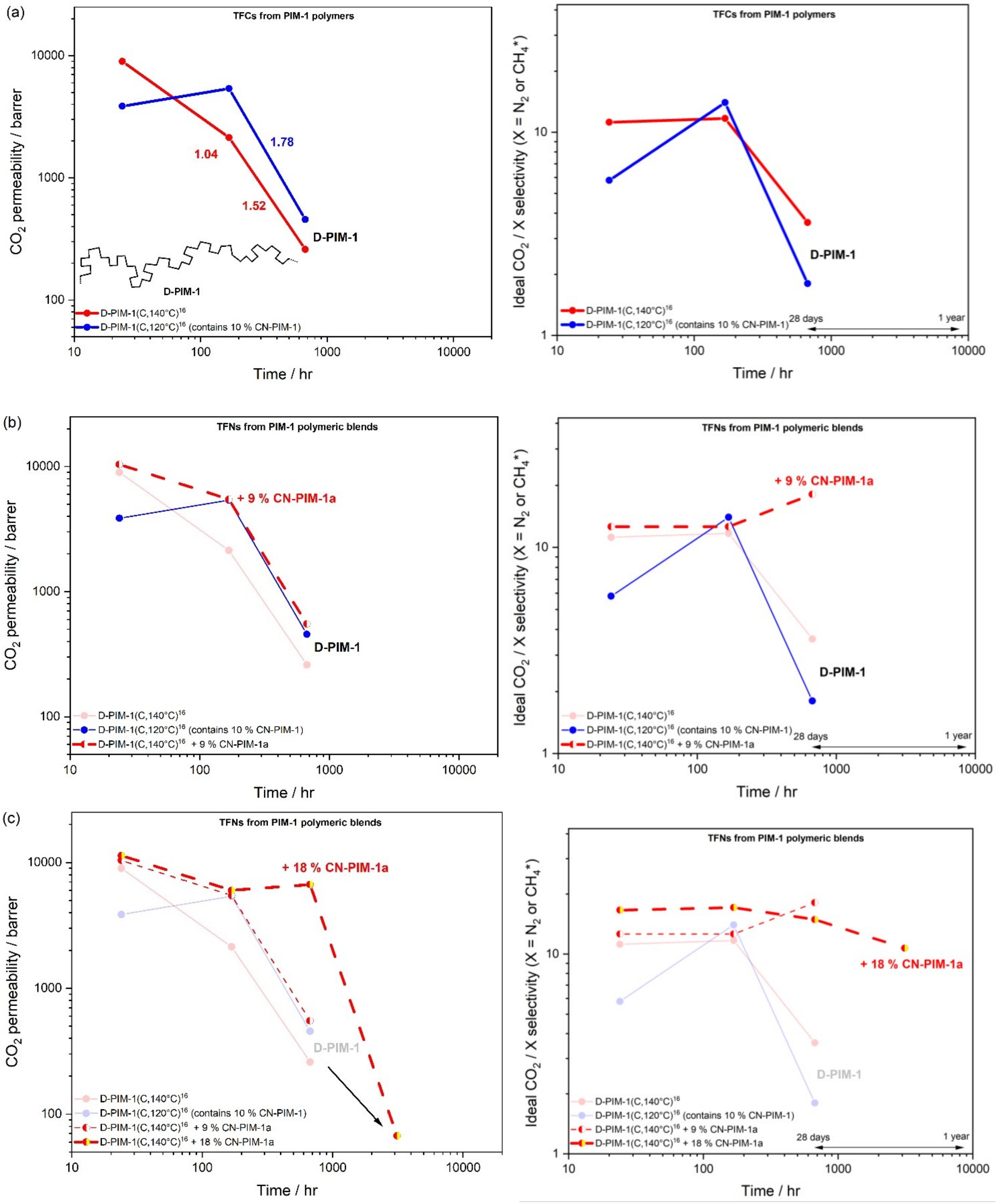

Figure S10.(a-c) Comparisons discussed within TFC and TFN aging data fabricated from D-PIM-1 polymer and their blends with network rich, CN-PIM-1a polymer presented in **Figure 4**.

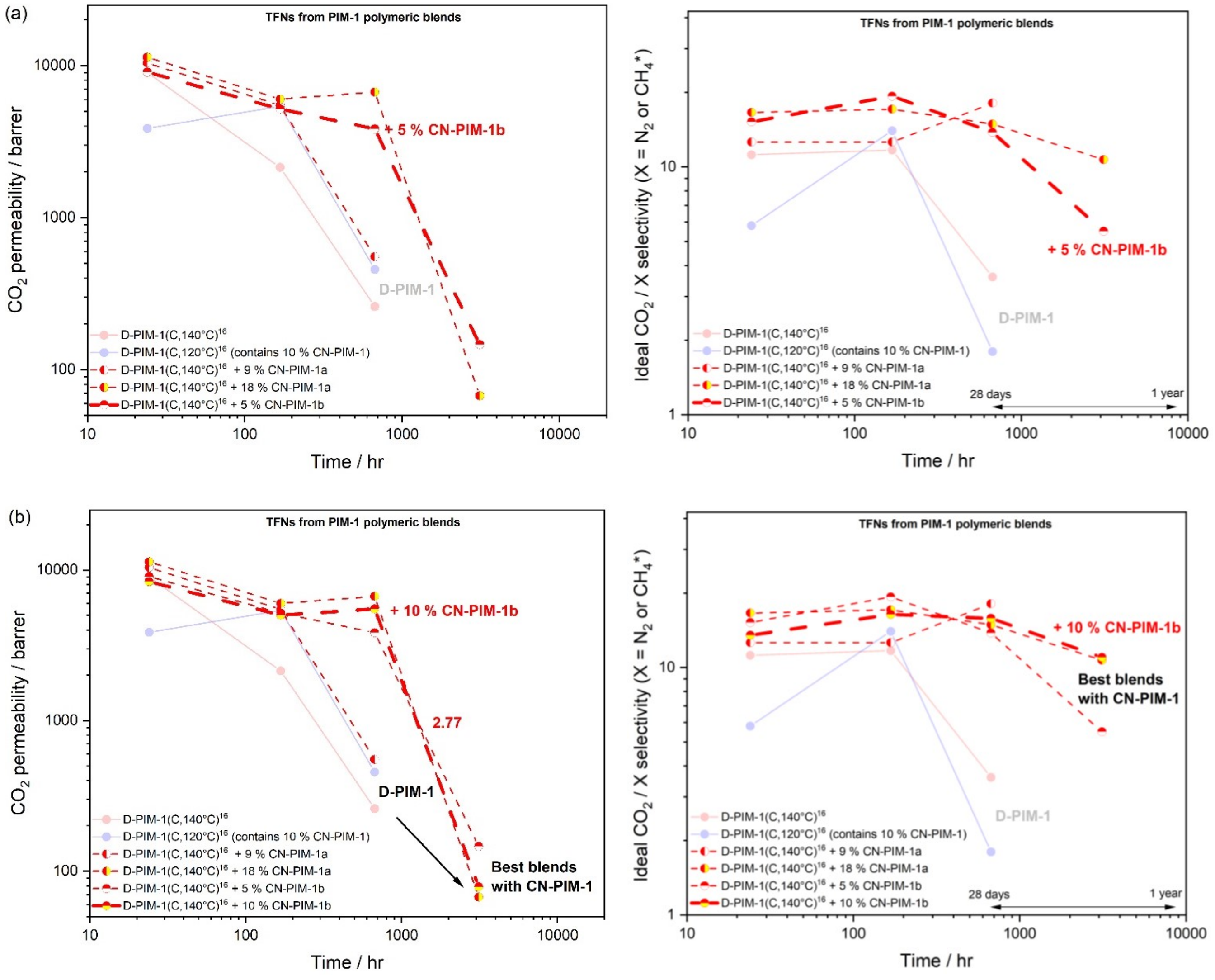

Figure S11.(a,b) Comparisons discussed within TFC and TFN aging data fabricated from D-PIM-1 polymer and their blends with network rich, CN-PIM-1b polymer presented in **Figure 4**.

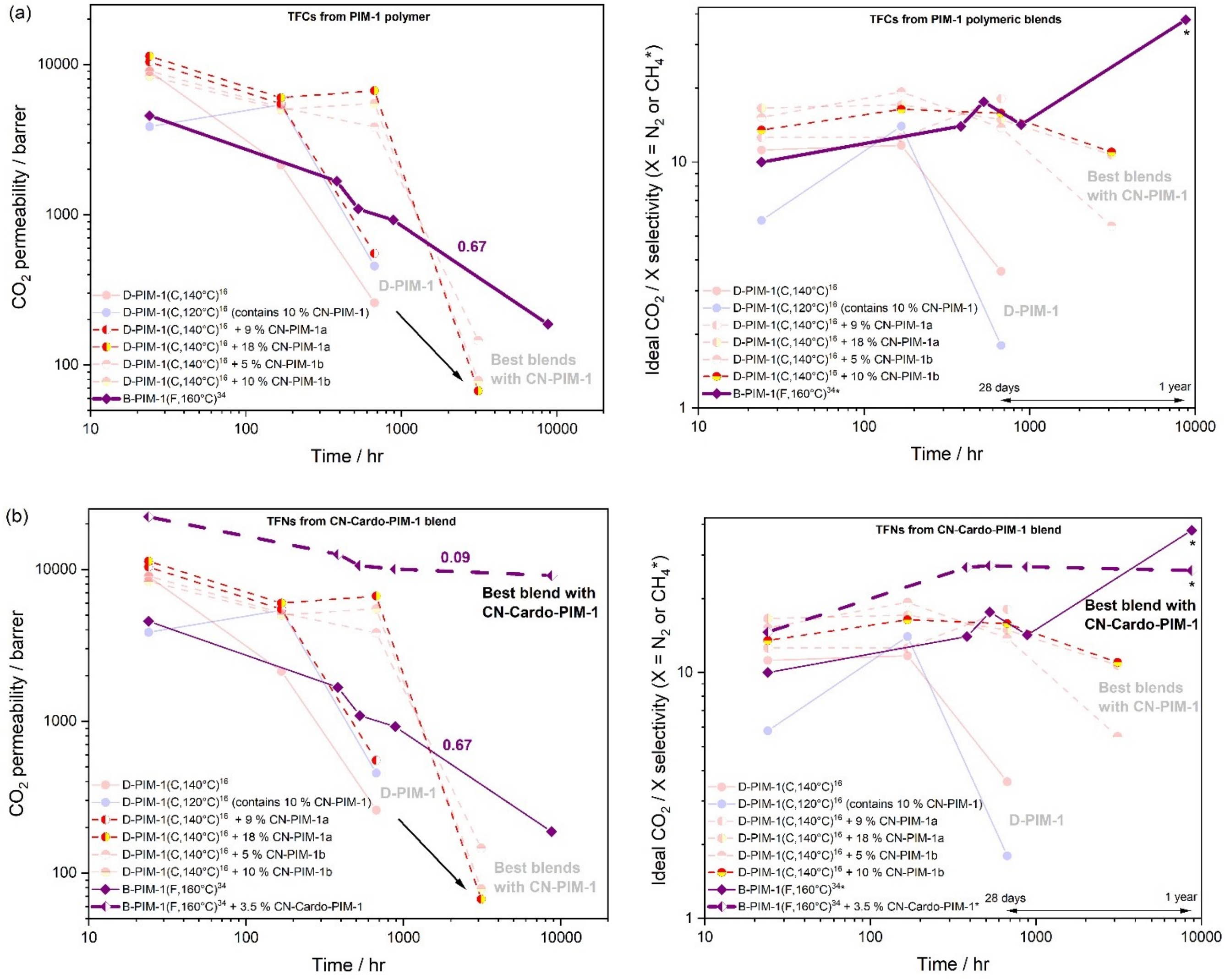

Figure S12.(a,b) Comparisons discussed within TFC and TFN aging data fabricated from B-PIM-1 polymer and blend with network rich, CN-Cardo-PIM-1 polymer presented in **Figure 4**.

S10. Permeability and ideal selectivity aging of PIM-1 based TFC and TFN membranes prepared since 2018.

Table S6. Permeability and ideal selectivity aging performance of kiss-coated PIM-1 TFCs, presented in **Figure 2**.

Ref.	Polymer	Fabrication			TFC performance				
		Support	Coating solution	Active layer / µm	Aging / day	$K(CO_2)$ / GPU	$P(CO_2)$ / barrer	CO_2 / N_2	CO_2 / CH_4
22	PIM-1(C,160 °C)[11]	PA350	3 % w/v $CHCl_3$	2.16	1	5079	10,970	12.4	-
		(Sepro)			7	1207	2610	13.6	-
					28	395	850	6.5	-
22	PIM-1(C,120 °C)[11]	PA350	3 % w/v $CHCl_3$	1.86	1	5985	11,130	19.9	-
		(Sepro)			7	1648	3070	20	-
					28	493	920	14.1	-
26	D-PIM-1(C,140 °C)[16]	PA350	3 % w/v $CHCl_3$	1.92	1	4678	8980	11.2	-
		(Sepro)			7	1114	2140	11.7	-
					28	134	260	3.6	-
26	D-PIM-1(C,120 °C)[16]	PA350	3 % w/v $CHCl_3$	1.33	1	2903	3860	5.8	-
		(Sepro)			7	4051	5390	14	-
					28	343	460	1.8	-
26	T-PIM-1(C,160 °C)[16]	PA350	3 % w/v $CHCl_3$	2.7	1	4642	12,630	13.3	-
		(Sepro)			7	1355	3690	30.4	-
					28	878	2390	27.6	-
					120	671	1830	26.7	-

Ref.	Polymer	Fabrication			TFC performance				
		Support	Coating solution	Active layer / µm	Aging / day	$K(CO_2)$ / GPU	$P(CO_2)$ / barrer	CO_2 / N_2	CO_2 / CH_4
[34]	B-PIM-1(C,120°C)[33]	PA350	3 % w/v $CHCl_3$	2.8	1	1583	4400	-	9.2
		(Sepro)			66	669	1860	-	23.4
					365	432	1200	-	12.3
[35]	B-PIM-1(F,160°C)[34]	PS35	3 % w/v $CHCl_3$	1.6	1	2850	4560	-	10
		(Solecta)			16	1045	1672	-	14
					22	682	1091	-	17.6
					37	575	920	-	14.2
					365	117	187	-	37.9
[28]	PIM-1(F,160°C)[18]	PS35	4.5 % w/v $CHCl_3$:THF (9:1)	2.0	1	4599	9198	21	13
		(Solecta)			7	2161	4322	23.8	17.4
					28	1331	2662	15.4	12.1
[32]	PIM-1(F,160°C)[23]	PA350	3 % w/v $CHCl_3$	2.5	1	3516	8790	12.4	6.2
		(Sepro)			7	1645	4113	19.6	11.6
					28	644	1610	22.4	16.0

Table S7. Permeability and ideal selectivity aging performance of kiss-coated PIM-1 TFCs and TFNs of blends with network-rich PIM polymers, presented in **Figure 4**.

Ref.	Polymer	Fabrication			TFC or TFN performance				
		Support	Coating solution	Active layer / µm	Aging / day	*K*(CO_2) / GPU	*P*(CO_2) / barrer	CO_2 / N_2	CO_2 / CH_4
26	D-PIM-1(C,140 °C)[16]	PA350	3 % w/v $CHCl_3$	1.92	1	4678	8980	11.2	-
		(Sepro)			7	1114	2140	11.7	-
					28	134	260	3.6	-
26	D-PIM-1(C,120 °C)[16] (10 % CN-PIM-1)	PA350	3 % w/v $CHCl_3$	1.33	1	2903	3860	5.8	-
		(Sepro)			7	4051	5390	14	-
					28	343	460	1.8	-
26	D-PIM-1(C,140°C)[16] + 9 % CN-PIM-1a	PA350	3 % w/v $CHCl_3$	1.92	1	5410	10,387	12.6	-
		(Sepro)			7	2850	5472	12.6	-
					28	287	551	18.1	-
26	D-PIM-1(C,140°C)[16] + 18 % CN-PIM-1a	PA350	3 % w/v $CHCl_3$	1.92	1	5910	11,347	16.6	-
		(Sepro)			7	3129	6008	17.1	-
					28	3480	6682	14.9	-
					130	35	67.2	10.7	-
26	D-PIM-1(C,140°C)[16] + 5 % CN-PIM-1b	PA350	3 % w/v $CHCl_3$	1.92	1	4708	9039	15.2	-
		(Sepro)			7	2697	5178	19.3	-
					28	1993	3827	13.8	-
					130	76	145.9	5.5	-

Ref.	Polymer	Fabrication			TFC or TFN performance				
		Support	Coating solution	Active layer / µm	Aging / day	$K(CO_2)$ / GPU	$P(CO_2)$ / barrer	CO_2 / N_2	CO_2 / CH_4
26	D-PIM-1(C,140°C)[16] + 10 % CN-PIM-1b	PA350	3 % w/v $CHCl_3$	1.92	1	4356	8364	13.5	-
		(Sepro)			7	2611	5013	16.4	-
					28	2871	5512	15.8	-
					130	41	78.7	11	-
35	B-PIM-1(F,160°C)[34]	PS35	3 % w/v $CHCl_3$	1.6	1	2850	4560	-	10.0
		(Solecta)			16	1045	1672	-	14
					22	682	1091	-	17.6
					37	575	920	-	14.2
					365	117	187	-	37.9
35	B-PIM-1(F,160°C)[34] + 3.5 % CN-Cardo-PIM-1	PS35	3 % w/v $CHCl_3$	1.76	1	12632	22,232	-	14.6
		(Solecta)			16	7157	12,596	-	26.8
					22	6018	10,592	-	27.2
					37	5703	10,037	-	26.9
					365	5193	9,140	-	26.0

Table S8. Permeability and ideal selectivity aging performance of PIM-1 TFCs and TFN blends with other fillers.

Ref.	Polymer	Fabrication			TFC or TFN performance				
		Support	Coating solution	Active layer / µm	Aging / day	$K(CO_2)$ / GPU	$P(CO_2)$ / barrer	CO_2 / N_2	CO_2 / CH_4
[17]	PIM-1 (F, 160 °C)[6]	PA350	3 % w/v $CHCl_3$	2.08	1	3331	6928	14.2	-
		(Sepro)			7	1441	2997	18.6	-
					30	826	1718	23.6	-
					50	562	1169	23.3	-
					90	388	807	24.5	-
[17]	PIM-1[6] + 60 wt % C-HCP	PA350	3 % w/v $CHCl_3$	8.05	1	27,530	n/a	6.6	
		(Sepro)			7	15,352	n/a	9.3	
					30	11,648	n/a	12.7	
					50	10,310	n/a	12.2	
					90	9379	n/a	11.3	
[34]	B-PIM-1(C,120°C)[33]	PS35	3 % w/v $CHCl_3$	2.8	1	1583	4400	-	9.2
		(Sepro)			66	669	1860	-	23.4
					365	432	1200	-	12.3
[34]	B-PIM-1[33] + 1 wt % HGO TAPA	PA350	3 % w/v $CHCl_3$	2.0	1	1050	2100	-	13.3
		(Sepro)			365	846	1692	-	12.8
[38]	PIM-1 (F, 160 °C)[40]	PA350	3 % w/v $CHCl_3$	3.25	1	2778	9029	-	6.3
		(Sepro)			7	201	653	-	6.3
					28	81	263	-	6.1

Ref.	Polymer	Fabrication			TFC or TFN performance				
		Support	Coating solution	Active layer / µm	Aging / days	*K*(CO_2) / GPU	*P*(CO_2) / barrer	CO_2 / N_2	CO_2 / CH_4
38	PIM-1[40] + 0.05 wt % S-SN	PA350	3 % w/v $CHCl_3$	2.25	1	3771	8485	-	8.7
		(Sepro)			7	1387	3121	-	9.6
					28	403	907	-	5.5
28	PIM-1(F,160°C)[18]	PS35	4.5 % w/v $CHCl_3$:THF (9:1)	2.0	1	4599	9198	21	13
		(Solecta)			7	2161	4322	23.8	17.4
					28	1331	2662	15.4	12.1
28	PIM-1[18] + 8.5 wt % C-UiO-66-NH_2/cPIM-1	PS35	4.5 % w/v $CHCl_3$:THF (9:1)	2.0	1	2664	5328	29.2	19.0
		(Solecta)			28	2559	5118	33.9	20.6
					63	2504	5008	37.2	23.8
27	PIM-1(F, 160 °C)[17]	UF010104	4 wt % THF	2.6	1	2000	5200	12.5	-
		(Solsep)			14	200	520	25.0	-
27	PIM-1[17] + 20 wt % PolyMOF	UF 010104	~4 wt % THF	2.7	1	4500	12,150	20.0	-
		(Solsep)			14	1125	3038	32.0	-
32	B-PIM-1(F,160°C)[23]	PA350	3 % w/v $CHCl_3$	2.5	1	3516	8790	12.4	6.2
		(Sepro)			7	1645	4113	19.6	11.6
					28	644	1610	22.4	16
32	B-cPIM-1 60 %[23]	PA350	4 % w/v THF	1.1	1	4671	5138	65.6	36.7
		(Sepro)			7	2038	2242	30.9	17.2

Ref.	Polymer	Fabrication			TFC or TFN performance				
		Support	Coating solution	Active layer / µm	Aging / days	$K(CO_2)$ / GPU	$P(CO_2)$ / barrer	CO_2 / N_2	CO_2 / CH_4
[32]	B-cPIM-1 60%[23]	PA350	4 % w/v THF	1.1	28	1483	1631	27.8	23.0
		(Sepro)							
[32]	B-cPIM-1 crosslinked with 3 mol % Co-MOF-74[23]	PA350	4 % w/v THF:methanol (10:1)	2.2	1	4297	9453	84.7	48.2
		(Sepro)			7	2223	4891	104.9	52.5
					28	717	1577	37.3	24.7

Table S9. Permeability and ideal selectivity aging performance of PIM-1 and cPIM-1 TFCs fabricated from THF solutions.

Ref.	Polymer	Fabrication			TFC performance				
		Support	Coating Solution	Active layer / µm	Aging / days	$K(CO_2)$ / GPU	$P(CO_2)$ / barrer	CO_2 / N_2	CO_2 / CH_4
32	B-cPIM-1 60 %[23]	PA350	4 % w/v THF	1.1	1	4671	5138	65.6	36.7
		(Sepro)			7	2038	2242	30.9	17.2
					28	1483	1631	27.8	23.0
11	D-PIM-1 (F, 160 °C)[19]	PA350	3 % w/v THF	3.72	1	6200	23,064	14	7.5
		(Sepro)			6	3000	11,160	20	12
					26	1800	6696	23	17
					60	930	3460	23	19
11	B-PIM-1 (F, 160 °C)[19]	PA350	3 % w/v THF	3.62	1	3100	11222	19	11
		(Sepro)			6	2600	9412	19	12
					20	2700	9774	19	12
					60	1100	3982	20	12
11	D-cPIM-1 70 %[19]	PA350	4 % w/v THF	0.63	1	7700	4851	56	37
		(Sepro)			6	4600	2898	130	91
					20	2400	1512	79	62
					60	3700	2331	40	28
11	B-cPIM-1 73 & 81 %[19]	PA350	4 % w/v THF	1.9	1	3200	6080	64	45
		(Sepro)		2.2	6	3100	6820	63	49
				1.9	20	3000	5700	76	55

Ref.	Polymer	Fabrication			TFC performance				
		Support	Coating Solution	Active layer / µm	Aging / days	*K*(CO_2) / GPU	*P*(CO_2) / barrer	CO_2 / N_2	CO_2 / CH_4
[11]	B-cPIM-1 73 & 81 %[19]	PA350 (Sepro)	4 % w/v THF	1.4	60	5000	7000	110	66
[11]	B-cPIM-1 90 %[19]	PA350 (Sepro)	4.75 % w/v THF	0.74	1	3300	2442	63	44
					6	1400	1036	49	43
					20	1200	888	59	45
					60	560	414	20	18
[11]	B-cPIM-1 90 %[19]	PA350 (Sepro)	6 % w/v THF	1.62	1	450	729	47	32
					6	350	567	68	34
					20	400	648	26	20
					60	790	1280	1.1	0.8

References

1. Song, Q.; Cao, S.; Pritchard, R. H.; Ghalei, B.; Al-Muhtaseb, S. A.; Terentjev, E. M.; Cheetham, A. K.; Sivaniah, E. Controlled thermal oxidative crosslinking of polymers of intrinsic microporosity towards tunable molecular sieve membranes. *Nat. Commun.* **2014**, *5*, 4813, DOI 10.1038/ncomms5813.
2. Kinoshita, Y.; Wakimoto, K.; Gibbons, A. H.; Isfahani, A. P.; Kusuda, H.; Sivaniah, E.; Ghalei, B. Enhanced PIM-1 membrane gas separation selectivity through efficient dispersion of functionalized POSS fillers. *J. Membr. Sci.* **2017**, *539*, 178-186, DOI 10.1016/j.memsci.2017.05.072.
3. Liu, M.; Lu, X.; Nothling, M. D.; Doherty, C. M.; Zu, L.; Hart, J. N.; Webley, P. A.; Jin, J.; Fu, Q.; Qiao, G. G. Physical aging investigations of a spirobisindane-locked polymer of intrinsic microporosity. *ACS Mater. Lett.* **2020**, *2* (8), 993-998, DOI 10.1021/acsmaterialslett.0c00184.
4. Chen, W.; Zhang, Z.; Hou, L.; Yang, C.; Shen, H.; Yang, K.; Wang, Z. Metal-organic framework MOF-801/PIM-1 mixed-matrix membranes for enhanced CO_2/N_2 separation performance. *Sep Purif Technol.* **2020**, *250*, 117198, DOI 10.1016/j.seppur.2020.117198.
5. Chen, W.; Zhang, Z.; Yang, C.; Liu, J.; Shen, H.; Yang, K.; Wang, Z. PIM-based mixed-matrix membranes containing MOF-801/ionic liquid nanocomposites for enhanced CO_2 separation performance. *J. Membr. Sci.* **2021**, *636*, 119581, DOI 10.1016/j.memsci.2021.119581.
6. Han, W.; Zhang, C.; Zhao, M.; Yang, F.; Yang, Y.; Weng, Y. Post-modification of PIM-1 and simultaneously in situ synthesis of porous polymer networks into PIM-1 matrix to enhance CO_2 separation performance. *J. Membr. Sci.* **2021**, *636*, 119544, DOI 10.1016/j.memsci.2021.119544.
7. Hou, R.; Smith, S. J. D.; Konstas, K.; Doherty, C. M.; Easton, C. D.; Park, J.; Yoon, H.; Wang, H.; Freeman, B. D.; Hill, M. R. Synergistically improved PIM-1 membrane gas separation performance by PAF-1 incorporation and UV irradiation. *J. Mater. Chem. A.* **2022**, *10* (18), 10107-10119, DOI 10.1039/D2TA00138A.
8. Sun, Y.; Geng, C.; Zhang, Z.; Qiao, Z.; Zhong, C. Two-dimensional basic cobalt carbonate supported ZIF-67 composites towards mixed matrix membranes for efficient CO_2/N_2 separation. *J. Membr. Sci.* **2022**, *661*, 120928, DOI 10.1016/j.memsci.2022.120928.
9. Begni, F.; Lasseuguette, E.; Paul, G.; Bisio, C.; Marchese, L.; Gatti, G.; Ferrari, M.-C. Hyper-Cross-Linked Polymers with Sulfur-Based Functionalities for the Prevention of Aging Effects in PIM-1 Mixed Matrix Membranes. *ACS Appl. Polym. Mater.* **2023**, *5* (6), 4011-4018, DOI 10.1021/acsapm.3c00246.
10. Guan, J.; Wang, X.; Du, J.; Liang, Q.; He, W.; Liu, Y.; Ma, J.; Zhang, C.; Liu, J. Surface-engineered PIM-1 membranes for facile CO_2 capture. *Chem. Eng. J.* **2023**, *477*, 147017, DOI 10.1016/j.cej.2023.147017.
11. Yu, M.; Foster, A. B.; Alshurafa, M.; Luque-Alled, J. M.; Gorgojo, P.; Kentish, S. E.; Scholes, C. A.; Budd, P. M. CO_2 separation using thin film composite membranes of acid-hydrolyzed PIM-1. *J. Membr. Sci.* **2023**, *679*, 121697, DOI 10.1016/j.memsci.2023.121697.
12. Chang, Q.; Guo, H.; Shang, Z.; Zhang, C.; Zhang, Y.; Dong, G.; Shen, B.; Wang, J.; Zhang, Y. PIM-based mixed matrix membranes containing covalent organic frameworks/ionic liquid composite materials for effective CO_2/N_2 separation. *Sep Purif Technol.* **2024**, *330*, 125518, DOI 10.1016/j.seppur.2023.125518.
13. Riaz, A.; Liu, L.; Xu, Z.; Liu, Q.; Younas, M.; Li, J.; Luo, C.; Ma, X. Nanocomposite membranes comprising covalent organic framework and polymer of intrinsic microporosity for efficient CO_2 separation. *Sep Purif Technol.* **2024**, *343*, 127175, DOI 10.1016/j.seppur.2024.127175.
14. Wang, K.; Chen, D.; Tang, J.; Hong, Z.; Zhu, Z.; Yuan, Z.; Lin, Z.; Liu, Y.; Semiat, R.; He, X. PIM-1-based membranes mediated with CO_2-philic MXene nanosheets for superior CO_2/N_2 separation. *Chem. Eng. J.* **2024**, *483*, 149305, DOI 10.1016/j.cej.2024.149305.

15. Khan, M. M.; Filiz, V.; Bengtson, G.; Shishatskiy, S.; Rahman, M.; Abetz, V. Functionalized carbon nanotubes mixed matrix membranes of polymers of intrinsic microporosity for gas saeparation. *Nanoscale Res. Lett.* **2012**, *7* (1), 504, DOI 10.1186/1556-276X-7-504.
16. Aliyev, E. M.; Khan, M. M.; Nabiyev, A. M.; Alosmanov, R. M.; Bunyad-Zadeh, I. A.; Shishatskiy, S.; Filiz, V. Covalently Modified Graphene Oxide and Polymer of Intrinsic Microporosity (PIM-1) in Mixed Matrix Thin-Film Composite Membranes. *Nanoscale Res. Lett.* **2018**, *13* (1), 359, DOI 10.1186/s11671-018-2771-3.
17. Bhavsar, R. S.; Mitra, T.; Adams, D. J.; Cooper, A. I.; Budd, P. M. Ultrahigh-permeance PIM-1 based thin film nanocomposite membranes on PAN supports for CO_2 separation. *J. Membr. Sci.* **2018**, *564*, 878-886, DOI 10.1016/j.memsci.2018.07.089.
18. Liang, C. Z.; Liu, J. T.; Lai, J.-Y.; Chung, T.-S. High-performance multiple-layer PIM composite hollow fiber membranes for gas separation. *J. Membr. Sci.* **2018**, *563*, 93-106, DOI 10.1016/j.memsci.2018.05.045.
19. Zhao, H.; Feng, L.; Ding, X.; Tan, X.; Zhang, Y. Gas permeation properties of a metallic ion-cross-linked PIM-1 thin-film composite membrane supported on a UV-cross-linked porous substrate. *Chin. J. Chem. Eng.* **2018**, *26* (12), 2477-2486, DOI 10.1016/j.cjche.2018.03.009.
20. Borisov, I.; Bakhtin, D.; Luque-Alled, J. M.; Rybakova, A.; Makarova, V.; Foster, A. B.; Harrison, W. J.; Volkov, V.; Polevaya, V.; Gorgojo, P.; et al. Synergistic enhancement of gas selectivity in thin film composite membranes of PIM-1. *J. Mater. Chem. A.* **2019**, *7* (11), 6417-6430, DOI 10.1039/c8ta10691f.
21. Putintseva, M. N.; Borisov, I. L.; Yushkin, A. A.; Kirk, R. A.; Budd, P. M.; Volkov, A. V. Effect of Casting Solution Composition on Properties of PIM-1/PAN Thin Film Composite Membranes. *Key Engineering Materials* **2019**, *816*, 167-173, DOI 10.4028/www.scientific.net/KEM.816.167.
22. Foster, A. B.; Tamaddondar, M.; Luque-Alled, J. M.; Harrison, W. J.; Li, Z.; Gorgojo, P.; Budd, P. M. Understanding the Topology of the Polymer of Intrinsic Microporosity PIM-1: Cyclics, Tadpoles, and Network Structures and Their Impact on Membrane Performance. *Macromolecules* **2020**, *53* (2), 569-583, DOI 10.1021/acs.macromol.9b02185.
23. Liu, M.; Nothling, M. D.; Webley, P. A.; Jin, J.; Fu, Q.; Qiao, G. G. High-throughput CO_2 capture using PIM-1@MOF based thin film composite membranes. *Chem. Eng. J.* **2020**, *396*, 125328, DOI 10.1016/j.cej.2020.125328.
24. Aliyev, E.; Warfsmann, J.; Tokay, B.; Shishatskiy, S.; Lee, Y. J.; Lillepaerg, J.; Champness, N. R.; Filiz, V. Gas Transport Properties of the Metal-Organic Framework (MOF)Assisted Polymer of Intrinsic Microporosity (PIM-1) Thin-Film Composite Membranes. *ACS Sustain Chem Eng.* **2021**, *9* (2), 684-694, DOI 10.1021/acssuschemeng.0c06297
25. Elsaidi, S. K.; Ostwal, M.; Zhu, L.; Sekizkardes, A.; Mohamed, M. H.; Gipple, M.; McCutcheon, J. R.; Hopkinson, D. 3D printed MOF-based mixed matrix thin-film composite membranes. *RSC Adv.* **2021**, *11* (41), 25658-25663, DOI 10.1039/d1ra03124d.
26. Foster, A. B.; Beal, J. L.; Tamaddondar, M.; Luque-Alled, J. M.; Robertson, B.; Mathias, M.; Gorgojo, P.; Budd, P. M. Importance of small loops within PIM-1 topology on gas separation selectivity in thin film composite membranes. *J. Mater. Chem. A.* **2021**, *9* (38), 21807-21823, DOI 10.1039/d1ta03712a.
27. Lee, T. H.; Lee, B. K.; Yoo, S. Y.; Lee, H.; Wu, W. N.; Smith, Z. P.; Park, H. B. PolyMOF nanoparticles constructed from intrinsically microporous polymer ligand towards scalable composite membranes for CO_2 separation. *Nat. Commun.* **2023**, *14* (1), 8330, DOI 10.1038/s41467-023-44027-y.
28. Qiu, B.; Yu, M.; Luque-Alled, J. M.; Ding, S.; Foster, A. B.; Budd, P. M.; Fan, X.; Gorgojo, P. High Gas Permeability in Aged Superglassy Membranes with Nanosized UiO-66−NH_2/cPIM-1 Network Fillers. *Angew. Chem. Int. Ed.* **2024**, *63* (1), e202316356, DOI 10.1002/anie.202316356.

29. Yu, M.; Foster, A. B.; Scholes, C. A.; Kentish, S. E.; Budd, P. M. Methanol Vapor Retards Aging of PIM-1 Thin Film Composite Membranes in Storage. *ACS Macro Lett.* **2023**, *12* (1), 113-117, DOI 10.1021/acsmacrolett.2c00568.
30. Lee, T. H.; Balçık, M.; Lee, B. K.; Ghanem, B. S.; Pinnau, I.; Park, H. B. Hyperaging-induced H_2-selective thin-film composite membranes with enhanced submicroporosity toward green hydrogen supply. *J. Membr. Sci.* **2023**, *672*, 121438, DOI 10.1016/j.memsci.2023.121438.
31. Zhou, Z.; Cao, X.; Lv, D.; Cheng, F. Hydrophobic metal–organic framework UiO-66-$(CF_3)_2$/PIM-1 mixed-matrix membranes for stable CO_2/N_2 separation under high humidity. *Sep Purif Technol.* **2024**, *339*, 126666, DOI 10.1016/j.seppur.2024.126666.
32. Alshurafa, M.; Foster, A. B.; Aloraini, S.; Yu, M.; Attfield, M. P.; Budd, P. M. Mixed matrix and thin-film nanocomposite membranes of PIM-1 and hydrolyzed PIM-1 with Ni- and Co-MOF-74 nanoparticles for CO_2 separation: Comparison of blending, grafting and crosslinking fabrication methods. *J. Membr. Sci.* **2025**, 713, 123388, DOI 10.1016/j.memsci.2024.123388.
33. Lee, T. H.; Jang, J. K.; Lee, B. K.; Wu, W.-N.; Smith, Z. P.; Park, H. B. Anomalous Structural Changes and Gas Transport Properties in Ultrathin Films of Polymers of Intrinsic Microporosity. *Macromolecules* **2024**, *57 (23)*, 11242-11250, DOI 10.1021/acs.macromol.4c01712.
34. Almansour, F.; Alberto, M.; Foster, A. B.; Mohsenpour, S.; Budd, P. M.; Gorgojo, P. Thin film nanocomposite membranes of superglassy PIM-1 and amine-functionalised 2D fillers for gas separation. *J. Mater. Chem. A.* **2022**, *10 (43)*, 23341-23351, DOI 10.1039/d2ta06339e.
35. Almansour, F.; Foster, A. B.; Ameen, A. W.; Mohsenpour, S.; Budd, P. M.; Gorgojo, P. High gas permeance in CO_2-selective thin film composite membranes from bis(phenyl)fluorene-containing blends with PIM-1. *J. Membr. Sci.* **2024**, *699*, 122652, DOI 10.1016/j.memsci.2024.122652.
36. Aloraini, S.; Mathias, M.; Crone, J.; Bryce, K.; Yu, M.; Kirk, R. A.; Ahmad, M. Z.; Asuquo, E. D.; Rico-Martínez, S.; Volkov, A. V.; et al. Crosslinking of Branched PIM-1 and PIM-Py Membranes for Recovery of Toluene from Dimethyl Sulfoxide by Pervaporation. *ACS Appl. Polym. Mater.* **2023**, *5* (2), 1145-1158, DOI 10.1021/acsapm.2c01600.
37. Devarajan, A.; Asuquo, E. D.; Ahmad, M. Z.; Foster, A. B.; Budd, P. M. Influence of Polymer Topology on Gas Separation Membrane Performance of the Polymer of Intrinsic Microporosity PIM-Py. *ACS Appl. Polym. Mater.* **2021**, *3* (7), 3485-3495, DOI 10.1021/acsapm.1c00415.
38. Mohsenpour, S.; Guo, Z.; Almansour, F.; Holmes, S. M.; Budd, P. M.; Gorgojo, P. Porous silica nanosheets in PIM-1 membranes for CO_2 separation. *J. Membr. Sci.* **2022**, *661*, 120889, DOI 10.1016/j.memsci.2022.120889.
